# Involvement of genes encoding ABI1 protein phosphatases in the response of *Brassica napus* L. to drought stress

**DOI:** 10.1007/s11103-015-0334-x

**Published:** 2015-06-10

**Authors:** Danuta Babula-Skowrońska, Agnieszka Ludwików, Agata Cieśla, Anna Olejnik, Teresa Cegielska-Taras, Iwona Bartkowiak-Broda, Jan Sadowski

**Affiliations:** Institute of Plant Genetics, Polish Academy of Sciences, Strzeszyńska 34, 60-479 Poznan, Poland; Department of Biotechnology, Institute of Molecular Biology and Biotechnology, Faculty of Biology, Adam Mickiewicz University, Umultowska 89, 61-614 Poznan, Poland; Plant Breeding and Acclimatization Institute – National Research Institute, Research Division in Poznań, Strzeszyńska 36, 60-479 Poznan, Poland

**Keywords:** *Brassica napus*, Drought response, ABI1 protein phosphatase, Gene overexpression, Promoter activity

## Abstract

In this report we characterized the *ArabidopsisABI1* gene orthologue and *Brassica napus* gene paralogues encoding protein phosphatase 2C (PP2C, group A), which is known to be a negative regulator of the ABA signaling pathway. Six homologous *B. napus* sequences were identified and characterized as putative PP2C group A members. To gain insight into the conservation of *ABI1* function in Brassicaceae, and understand better its regulatory effects in the drought stress response, we generated transgenic *B. napus* plants overexpressing *A. thalianaABI1*. Transgenic plants subjected to drought showed a decrease in relative water content, photosynthetic pigments content and expression level of *RAB18*- and *RD19A*-drought-responsive marker genes relative to WT plants. We present the characterization of the drought response of *B. napus* with the participation of *ABI1*-like paralogues. The expression pattern of two evolutionarily distant paralogues, *BnaA01.ABI1.a* and *BnaC07.ABI1.b* in *B. napus* and their promoter activity in *A. thaliana* showed differences in the induction of the paralogues under dehydration stress. Comparative sequence analysis of both *BnaABI1* promoters showed variation in positions of *cis*-acting elements that are especially important for ABA- and stress-inducible expression. Together, these data reveal that subfunctionalization following gene duplication may be important in the maintenance and functional divergence of the *BnaABI1* paralogues. Our results provide a framework for a better understanding of (1) the role of ABI1 as a hub protein regulator of the drought response, and (2) the differential involvement of the duplicated *BnaABI1* genes in the response of *B. napus* to dehydration-related stresses.

**Electronic supplementary material:**

The online version of this article (doi:10.1007/s11103-015-0334-x) contains supplementary material, which is available to authorized users.

## Introduction

Drought stress induces diverse responses at the cellular and molecular levels including the production of plant phytohormones, especially abscisic acid (ABA) (Swamy and Smith 1999; Zhu 2003). The core ABA signaling pathway consists of three important elements: ABA receptors (PYR/PYL/RCAR proteins), protein phosphatases type 2C (PP2Cs) and SNF1-related protein kinases (SnRK2s) (Yoshida et al. 2006; Ma et al. 2009; Fujita et al. 2009, Umezawa et al. 2010). In *Arabidopsis*, there are nine group A PP2Cs: ABI1, ABI2, HAB1, HAB2, HAI1, HAI2, HAI3, AHG1 and AHG3 (Gosti et al. 1999; Merlot et al. 2001; Yoshida et al. 2006; Rubio et al. 2009). It is widely accepted that group A PP2Cs have a key role in the ABA signaling pathway (Sheen 1998; Schweighofer et al. 2004; Ludwików 2015 for review). ABA stimulates plant responses to abiotic stresses such as drought, high salt and cold, and several research teams have shown that group A PP2Cs, including ABI1 (ABA Insensitive 1; Leung et al. 1994), are negative regulators of ABA signaling (Gosti et al. 1999; Merlot et al. 2001; Saez et al. 2004). Although it is known that PP2Cs interact with various targets to form a regulatory core (Ludwików 2015 for review) that modulates cellular processes and stress response pathways (Nemhauser et al. 2006; Ludwików et al. 2009; Umezawa et al. 2009; Geiger et al. 2010; Ludwików et al. 2014), their role in the acclimation of plants to drought stress remains unclear. Hence it is important to investigate further the function of ABI1 and other group A PP2Cs in the stress responses of plants, including economically important crops with complex genomes typically carrying multiple gene homologues.

Although group A PP2Cs have been extensively analyzed in *Arabidopsis*, their orthologues (ancestrally related genes maintained in different species after speciation events) have also been identified and partially characterized in other plant species (Gonzalez-Garcia et al. 2003; Xue et al. 2008; Tougane et al. 2010; Ludwików et al. 2013; Yuan et al. 2013, Jia et al. 2013). In *Brassica* species, protein-encoding genes are generally triplicated relative to their counterparts in *A. thaliana* due to whole-genome duplication events (Babula et al. 2003; Parkin et al. 2005; Lysak et al. 2005; Babula et al. 2006; Ziolkowski et al. 2006; Wang et al. 2011) between 7.9 and 14.6 Mya. Despite the high degree of sequence similarity of their transcripts and the corresponding proteins, such paralogous genes often exhibit significant differences in expression patterns. This can result in increased overall gene expression levels and can provide a source of novel gene variants (Udall and Wendel 2006; Whittle and Krochko 2009). The potential for functional divergence among duplicated genes and consequent pleiotropic effects on stress responses, especially to stresses such as drought, is poorly described for crop plants including the *Brassica* species.

Currently, there is a paucity of information on the role of paralogous *ABI1* copies (they are considered in-paralogues, i.e. they result from duplication after the last speciation event; Studer and Robinson-Rechavi 2009) in the regulation of the ABA signaling pathway in the *Brassica* species. Previous studies have focused on individual *ABI1* gene copies in particular species, and have neglected to investigate paralogous examples (Yuan et al. 2013; Zhang et al. 2014).

In this study we have investigated the conservation of the role of the *ABI1* gene orthologue in Brassicaceae (*Brassicanapus* vs. *Arabidopsis*) and the duplicated *ABI1* genes in *B. napus* with particular reference to the response to drought stress. We generated transgenic *B. napus* plants overexpressing the *A. thalianaABI1* orthologue to study corresponding changes in the drought-stress response, as well as the cumulative effect of all *BnaABI1*-like genes under drought conditions. Overexpression of *AtABI1* resulted in water loss and changes in chlorophyll accumulation indicating that these stress-related physiological responses are controlled by the ABI1-dependent ABA-signaling pathway. Transcript levels of known drought stress marker genes were also downregulated in transgenic plants compared to wild type *B. napus*. We also observed changes in the transcript levels of the endogenous *ABI1* homologues (*BnaABI1* *s*) in wild type (WT) and transgenic *B. napus* to evaluate their involvement in the drought response. This aspect presents important insight into poorly understood dehydration-induced co-response of duplicated gene homologues occurring typically in plants. To determine whether the duplicated *BnaABI1* genes are functionally redundant or whether they have evolved novel subfunctions, we performed a detailed analysis of two evolutionarily distant *BnaABI1* paralogues, *BnaA01.ABI1.a* and *BnaC07.ABI1.b*, and found evidence for structural and functional diversification. We demonstrated that the promoter activity of these paralogues in *A. thaliana* was sufficient to control tissue-specific expression, but resulted in different stress-induced expression. The combined results shed new light on diverse roles of the *ABI1* gene family in the drought response plasticity in the *Brassica* polyploid. In-depth knowledge about the functional divergence of *BnaABI1* members will facilitate future work on genetic engineering of *Brassica* species.

## Materials and methods

### Plant material, growth and stress conditions

*Arabidopsis thaliana* (L.) Heynh. ecotype Columbia (Col-0) and *B. napus* microspore-derived embryos of cv. Monolit (HR Strzelce, Poland) were used to generate homozygous transgenic plants. Plant growth conditions, exogenous ABA and drought stress treatments were described previously by Ludwików et al. (2013). The *B. napus* seeds were germinated in sterilized petri dishes on sand for 3 days at 25 °C. After incubation, good quality seedlings with fully expanded green cotyledon were grown in pots (7 cm × 7 cm × high 8 cm; 2 seedlings per pot; 20 pots per condition) filled with soil in a growth room under long-day conditions (16/8 h of light/dark with 18 °C). They were watered every 2 days for 3 weeks and fertilized weekly with Florovit (0.5 ml/L). The drought stress was induced in well-watered three-week-old plants by withholding water for 5 days. These plants were then re-watered for 5 days after which leaves were collected. In parallel, control plants received water regularly. Relative water content (RWC) was calculated according to the formula: [(fresh weight − dry weight)/(turgid weight − dry weight)] × 100. For wounding treatment all rosette leaves were crushed with forceps, which effectively wounded 40 % of the leaf area (Reymond et al. 2000). All measurements were made on three or more replicates.

### Identification and cloning of *ABI1* paralogues from the *B*. *napus* genome

The *A. thaliana ABI1* cDNA sequence (At4g26080) was used as a reference sequence to detect the corresponding *B. napus* EST and GSS clones. The respective *B. napus* clone collections were screened by BLASTN search against the ATiDB (The *Arabidopsis thaliana* Integrated Database) and NCBI (www.ncbi.nlm.nih.gov) databases. The selected EST sequences were used to clone *ABI1*-related genes with gene-specific primer sets and *B. napus* genomic DNA as the template. Genomic DNA was extracted using a DNeasy Plant Mini kit (Qiagen). For full-length cDNA amplification, the Rapid Amplification of cDNA Ends (RACE) method was used using a SMARTer™ RACE cDNA Amplification Kit (Clontech) according to the manufacturer’s instructions. All primer sequences are listed in Supplemental Table S1. The resulting PCR amplicons for genomic and full length cDNA sequences corresponding to individual paralogues were subcloned into StrataClone™ PCR Cloning Vector pSC-A (Agilent Technologies) and confirmed by sequencing. The NCBI Conserved Domain Database (http://www.ncbi.nlm.nih.gov/Structure/cdd/wrpsb.cgi) was used to identify the PP2C catalytic domain and characteristic motifs within deduced BnaABI1 protein sequences.

### Phylogenetic analysis of *ABI1*-related genes and other group A PP2Cs in *A. thaliana* and *B. napus*

The deduced group A PP2C amino acid sequences of *B. napus* (Brassica Database; http://brassicadb.org/brad/) were used to estimate their phylogenetic relationships. The clone numbers corresponding to individual PP2C are listed in Supplemental Table S2. First, full-length protein sequences were aligned using the ClustalX program with default parameters. Selected amino acid sequences of the conserved PP2C catalytic domain were further imported into MEGA5.1 software (http://www.megasoftware.net/; Tamura et al. 2011). The phylogenetic tree was constructed using the neighbor-joining method with a Poisson model of amino acid substitution and 1000 bootstrap replications.

### Plasmid construction for *B. napus* transgenic plants overexpressing *AtABI1*

For *B. napus* transformation, the coding region of the *AtABI1* gene (At4g26080) was PCR-amplified using gene-specific primer pairs (Table S1). The amplified *AtABI1* coding sequence was cloned into the pKGIB vector between the *Bam*HI and *Xho*I sites under the control of a 35S-CaMV promoter (Cegielska-Taras et al. 2008). The construct was introduced into *B. napus* via *Agrobacterium*-mediated transformation using the method described by Cegielska-Taras et al. (2008). Putative plant transformants were screened on 1/2 MS media including 10 mg/L phosphinotricin. T2 homozygous plants were used for further analysis.

### Measurements of physiological parameters

The chlorophyll and carotenoid contents were determined as described in Ludwików et al. (2009). Ten plants per line were evaluated and all tests were repeated three times.

### RNA isolation and qPCR analysis

RNA isolation and quantitative real-time PCR (qPCR) was performed as described by Ludwików et al. (2013). Total RNA was extracted from three-week old leaves of the WT and transgenic plants using TRIZOL reagent (Invitrogen) following the manufacturer’s instructions. For quantitative RT-PCR analysis, 1.5 μg DNase-treated RNA was used for the first-strand cDNA synthesis using a First Strand cDNA Synthesis Kit (Fermentas, Gdańsk, Poland) according to manufacturer’s protocol. 2 μL of 10-fold diluted cDNA was used as the template in a 15 μL qRT-PCR reaction using Maxima SYBR Green/ROX/qPCR Master Mix (Fermentas). An 18S rDNA gene was used as the internal control to normalize all data. Each sample was set up with three biological replicates and run twice on separate plates. PCR reactions were conducted in the ABI PRISM 7900 HT sequence detection system (Applied Biosystems). The qRT-PCR amplifications were carried out using the following thermal profile: 50 °C for 2 min, 95 °C for 10 min, followed by 40 cycles of 95 °C for 15 s and 60 °C for 1 min. Data analysis was performed using SDS 2.2.1 software (Applied Biosystems). Serial dilutions of genomic DNA (from 100 to 10,000 copies) were used to set up the calibration curve. The results presented here are the mean of the relative gene expression ratios [log2 fold change scale; FC = log2 (ratio/ratio)] of two independent experiments (n = 6). After the PCR, a melting curve was generated to test the amplicon specificity. Primer sequences are listed in Supplemental Table S1.

### Promoter activity assay

The regions almost 2.2 kb upstream of the translation start codon for both the *BnaA01.ABI1.a* and *BnaC07.ABI1.b* genes were isolated using the GenomeWalker kit (Clontech) following the manufacturer’s instructions. Gene-specific primers for the nested PCR are listed in Supplemental Table S1. For the promoter activity assay, the promoter sequences of the selected *BnaABI1* paralogues were subcloned into the pENTR/SD/TOPO cloning vector and confirmed by sequencing. To produce plant transformants, pENTR vector-promoter constructs were recombined with the pMDC162 (::GUS) vector using *Gateway*^®^ LR *Clonase*^®^ II Enzyme Mix (Invitrogen). The promoter-reporter fusion construct was transformed into *Agrobacterium tumefaciens* strain LBA4404 and used for *A. thaliana* Columbia line plant transformations using the floral dip method (Clough and Bent 1998). Putative plant transformants were screened on 1/2 MS media including 10 mg/L hygromycin. For histochemical analysis of *GUS* expression, tissue samples were immersed in GUS staining solution (100 mM sodium phosphate, pH 7.0, 10 mM EDTA, 0.5 mM K_4_Fe[CN]_6_, 0.5 mM K_3_Fe[CN]_6_, 0.1 % Triton X-100 and 1 mM X-gluc) overnight at 37 °C. After staining, plant samples were rinsed and photographed.

### Identification of *BnaA01.ABI1.a* and *BnaC07.ABI1.bcis*-regulatory elements

Potential regulatory elements were identified within the 2,180 bp upstream of the *BnaA01.ABI1.a* and *BnaC07.ABI1.b* start codons using the MathInspector platform (Genomatix; http://www.genomatix.de/) with the Plant IUPAC Library Version 7.0 (based on PLACE Release 30.0) restricted to *A. thaliana* and IUPAC search parameters and max. 0 % mismatches. Additionally, stress-responsive regulatory motifs were identified by searching the publicly available *cis*-acting element databases such as Plant Matrix Family Library (Genomatix) and PlantCare (http://bioinformatics.psb.ugent.be/webtools/plantcare/html/). “Conserved Non-Coding Sequences”, named “CNSs”, including conserved transcription factor binding sites within the *BnaA01.ABI1.a* and *BnaC07.ABI1.b* promoters, were defined by sequence similarity (>70 % identity over at least 90 bp). Furthermore, these highly conserved regions were analyzed for the presence of conserved stress-responsive *cis*-regulatory elements using Plant IUPAC Library Version 7.0 on the MathInspector platform. Regions conserved between both promoters were visualized using VISualization Tool for Alignments (Vista) tools mVista and rVista (http://rvista.dcode.org/).

## Results

### Response of *AtABI1*-overexpressing transgenic *B. napus* lines to drought stress: effect on RWC and photosynthetic pigment levels

**Fig. 1:**
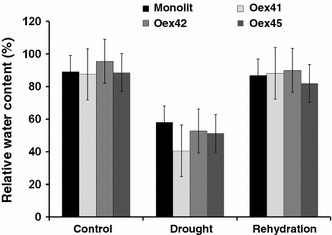
Relative water content (RWC) in *B. napus* wild-type and transgenic plants during drought and rehydration. The data represent means from five replicates with three biological repeats. *Error bars* represent SE

To investigate the potential conservation of ABI1 protein phosphatase 2C function in *B. napus,AtABI1*-overexpressing *B. napus* lines under the control of a 35S-CaMV promoter were generated. Six independent transgenic plants were produced in which the presence of the transgene was confirmed by PCR assay with *NOSt/BAR*-specific primers (not shown). Three representative lines containing the *35S*:*AtABI1* construct were used in subsequent experiments (Fig. S1). The transgenic plants were not morphologically different to wild type plants under normal growth conditions (not shown). Because of the involvement of the *ABI1* gene in the abiotic stress response (Leung et al. 1997; Chinnusamy et al. 2004), the effect of *AtABI1* overexpression in *B. napus* plants during drought and rehydration was analyzed. Water status (determined as RWC. i.e. relative water content in leaves) and changes in photosynthetic pigment content were examined in both wild-type plants and transgenic lines grown under well-watered and water-deficient conditions. Measurements of water loss showed no significant differences in RWC between the *B. napus* control and transgenic plants under normal growth conditions (Fig. 1). However, water content in the leaves of transgenic plants decreased more rapidly during soil drying compared to wild-type plants. In particular, drastic water loss in the leaves of transgenic lines Oex41 (Overexpression line 41) and Oex42 were observed, where RWC decreased by at least 40 %. This demonstrates that overexpression of the *AtABI1* orthologue in *B. napus* plants reduces their water retention capacity, confirming ABI1 role as ABA negative regulator.

**Fig. 2:**
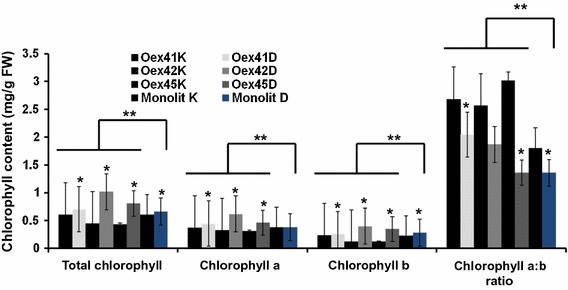
Total chlorophyll content, chlorophyll *a*, chlorophyll *b* and chlorophyll *a*:*b* ratio in wild type and *ABI1*-overexpressing transgenic plants under drought conditions. K: control (well-watered) conditions, D: drought conditions. The data represent means from five replicates with three biological repeats. * indicates *P* < 0.05 between control and experiment (drought); ** indicates *P* < 0.05 between the wild type (Monolit) and the *ABI1*-overexpressing lines by *t* test. *Error bars* indicate SE

These data were complemented by assessment of the content of photosynthetic pigments such as chlorophyll and carotenoids in these plants under well-watered and drought-stress conditions. Initially, chlorophyll *a*, chlorophyll *b*, total chlorophyll content and chlorophyll *a*/*b* ratio were analyzed. Changes in photosynthetic pigment content were observed in both wild-type plants and transgenic lines under drought-stress conditions (Fig. 2). As expected, chlorophyll *a*, chlorophyll *b* and total chlorophyll contents significantly increased in all *B. napus* transgenic lines overexpressing *AtABI1*. Chlorophyll *a* levels were higher in all transgenic plants than in wild-type plants, while remaining unchanged in wild-type plants under both drought-stress and control conditions. Chlorophyll *b* content remained almost invariable in transgenic line Oex41 and in wild-type plants, while its content in lines Oex42 and Oex45 increased and was higher than in wild-type plants. In general, chlorophyll *a* levels were higher than chlorophyll *b* levels in both types of plant. However, the chlorophyll *a*/*b* ratio decreased in both wild-type plants and the transgenic lines under drought-stress conditions. The largest decrease in chlorophyll *a*/*b* ratio was observed in transgenic line Oex45.

**Fig. 3:**
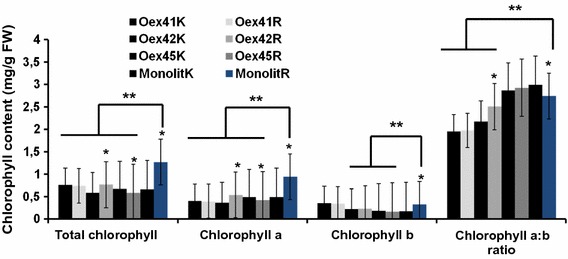
Total chlorophyll content, chlorophyll *a*, chlorophyll *b* and chlorophyll *a*:*b* ratio in the wild type (Monolit) and *ABI1*-overexpressing plants after rehydration. K: control (well-watered) conditions, R: drought-treated samples after rehydration. The data represent means from five replicates with three biological repeats. * indicates *P* < 0.05, between control and experiment (rehydration); ** indicates *P* < 0.05 between wild type and *ABI1*-overexpressing lines by *t* test. *Error bars* indicate SE

Less variation in the photosynthetic pigment content was observed during rehydration conditions (Fig. 3), but there were, nevertheless, slight differences between particular *B. napus* transgenic lines overexpressing *AtABI1*. Line Oex41 showed no changes in chlorophyll *a*, chlorophyll *b* and total chlorophyll contents rehydration compared to controls. Chlorophyll *b* content was invariable in all transgenic lines, but increased in wild type plants under rehydration compared to control, well-watered conditions. On the other hand, total chlorophyll and chlorophyll *a* contents increased and slightly decreased in lines Oex42 and Oex45, respectively. The chlorophyll *a*/*b* ratio increased slightly in all transgenic lines and decreased in the wild-type plants on rehydration.

**Fig. 4:**
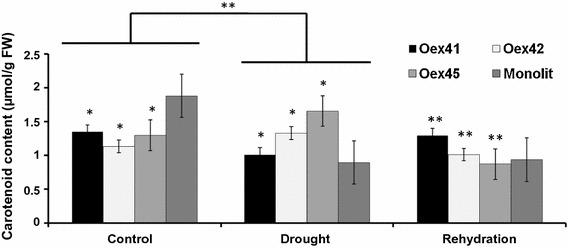
Total carotenoid content of the wild-type (Monolit) and 35S:*ABI1* lines under drought and rehydration conditions. K: control (well-watered) conditions, D: drought conditions. The data represent means from five replicates with three biological repeats. ** indicates *P* < 0.05, between control and experiment (drought); * indicates *P* < 0.05 between wild type and *ABI1*-overexpressing lines by *t* test. *Error bars* indicate SE

Analysis of carotenoid content during drought stress showed a decrease in both wild-type plants and line Oex41, but an increase in lines Oex42 and Oex45 compared to their respective controls (Fig. 4). On rehydration, carotenoid content returned to the control level in transgenic lines Oex41 and Oex42, but decreased in line Oex45 and wild type plants.

### Expression of selected ABA-responsive and drought-responsive genes in WT and transgenic *ABI1*-overexpressing lines of *B. napus*

**Fig. 5:**
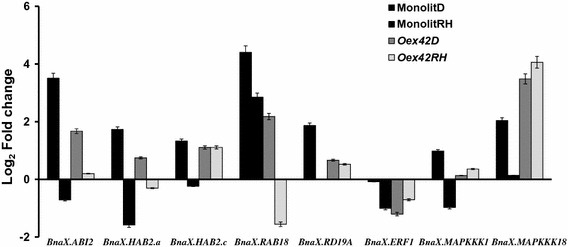
The relative expression of selected ABA-responsive and drought-responsive genes in wild-type plants and *AtABI1*-overexpressing *B. napus* transgenic lines during drought and after rehydration. The qPCR results show relative expression of the indicated genes. Gene transcript levels were determined using three replicates and were normalized against 18S rDNA. Each quantification was repeated at least twice with similar results. The results are displayed as mean log2 fold change ± SE (n = 9) of three independent experiments

A set of ABA- and/or stress-inducible genes, as well as other components of the ABA signaling pathway selected from publicly available microarray data for *A. thaliana* (https://www.genevestigator.com/gv/plant.jsp), were included in our analysis. Among them were genes reported to be inducible by exogenous ABA and/or drought such as *RAB18*, *RD19A* and *MAPKKK18*, other members of the PP2C gene family such as *ABI2*, two copies of *HAB2* and genes induced by other factors such as *MAPKKK1* (cold and heat responsive) and *ERF1* (ethylene responsive). As shown in Fig. 5 for wild type plants and representative transgenic line Oex42, the transcript levels of all genes with the exception of *BnaX.ERF1*, *BnaX.HAB2.c* and *BnaX.MAPKKK1* increased significantly in wild-type plants and line Oex42 overexpressing *AtABI1* in response to drought stress. However, these genes with the exception of *BnaX.MAPKKK18* were expressed at a low level in line Oex42. The expression level of most of these genes returned to control values in both wild-type and transgenic plants under rehydration conditions. Interestingly, the two copies of *BnaX.HAB2* studied showed different expression profiles, with only one copy being significantly induced under drought stress. This indicates functional divergence of the duplicated *BnaHAB2* genes as an example of gene subfunctionalisation in the ABA signaling network.

Taken together, these results confirmed that *BnaABI1* genes are negative regulators of the drought response in *B. napus*. Overexpression of *AtABI1* in the *B. napus* transgenic lines results in decreased tolerance to drought stress.

### Structural characteristics and phylogenetic analysis of the *ABI1* paralogues in *B*. *napus*

Using the *ArabidopsisAtABI1* cDNA to query the ATiDB and NCBI databases, six *ABI1*-related genes were identified in the amphidiploid genome of *B. napus*. They are paralogous *ABI1* copies, which result from the duplication after last speciation event. These six *BnaABI1* family members were cloned using paralogue-specific primers and then verified by sequencing (Table S1). They were named according to the nomenclature system proposed for the *Brassica* genus (Østergaard and King 2008). All *BnaABI1*-related genes were structurally characterized and their chromosomal localization in the *B. napus* genome was determined (Babula-Skowronska et al., unpublished). To confirm homology between the *ABI1* family members in *B. napus* and their diploid *B. rapa* and *B.oleracea* progenitors, a phylogenetic tree was built with high bootstrap support (Fig. S2). This analysis was performed using amino acid sequences of the conserved PP2C catalytic domain for six, three and three ABI1 sequences from *B. napus*, *B. rapa* and *B. oleracea*, respectively. Additionally, the previously detected ABI1s from *B. oleracea* (KF577723, corresponding to *BolC08.ABI1.c*; Yuan et al. 2013) and *B. napus* (JX122895, corresponding to *BnaC01.ABI1.a*; Zhang et al. 2014) were included. As expected, there was a high degree of similarity between the *ABI1* orthologues derived from the *B. napus*, *B. rapa* and *B. oleracea* genomes, while the paralogues within the same genomes of the individual species showed greater sequence divergence. A phylogenetic analysis of group A PP2Cs of *A. thaliana* and *B. napus* also showed that they divided into two subgroups with high bootstrap support. Moreover, the close evolutionary relationship between the *BnaA01.ABI1.a* and *AtABI1* genes was confirmed based on protein sequence alignment of the PP2C catalytic domain (Fig. S3). These results suggest close evolutionary relationships between the respective *A. thaliana* and *B. napus* genes.

To investigate the possibility of a functional plasticity of the duplicated *BnaABI1* gene copies in response to environmental stress conditions, two evolutionarily distant *BnaABI1* genes, *BnaA01.ABI1.a* and *BnaC07.ABI1.b,* were chosen for subsequent experiments. The first of these represents the closest homologue to *A. thalianaAtABI1*, whereas the second is a more distant homologue of *AtABI1*, dating from the whole-genome duplication that took place after the *Arabidopsis* and *Brassica* lineages diverged (Fig. S2). By screening *B. rapa* (donor of A genome) and *B. oleracea* (donor of C genome) genomic DNA using *BnaABI1* paralogue-specific primers, *BnaA01.ABI1.a* and *BnaC07.ABI1.b* were assigned to chromosome A01 in the A genome and chromosome C07 in the C genome, respectively (Fig. S4). Detailed analysis of both *BnaABI1* genomic and cDNA sequences revealed a highly conserved gene structure with four exons and three introns (Fig. S5). The lengths of the *BnaA01.ABI1.a* and *BnaC07.ABI1.b* genomic sequences are 1588 and 1519 bp, coding sequences are 1281 and 1233 bp and deduced protein sequences are 426 and 410 aa, respectively. The sequence similarity of both *BnaABI1* paralogues ranges from 78.6 up to 84.4 % at the genomic and coding sequence levels, respectively. Major sequence differences between both *BnaABI1* genes were discovered in the 5′-UTR, first exon and all introns (Fig. S6). As shown in Fig. S6, the most frequent sequence changes between the two *BnaABI1* genes are deletion/insertion (indels) of longer fragments. For example, one of the longer indels, which includes a microsatellite motif (CAT), is located in the first exon; additional differences occur in sequences flanking this repeat motif. However, the lengths of exon II, exon III and exon IV, 291, 106 and 359 bp, respectively, were maintained in both *BnaABI1* paralogues. To confirm that the selected *BnaABI1* genes belong to the PP2C-type phosphatase gene family, the presence of the PP2C catalytic domain in the deduced protein sequences was determined. Both BnaABI1 proteins contained the PP2C catalytic domain at the C-terminus with the same arrangement of 11 motifs; the N-terminal noncatalytic region was variable in both proteins (Figs. S3 and S7).

### Expression of *ABI1*-related endogenes in transgenic *ABI1*-overexpressing and WT lines of *B. napus* exposed to drought stress

**Fig. 6:**
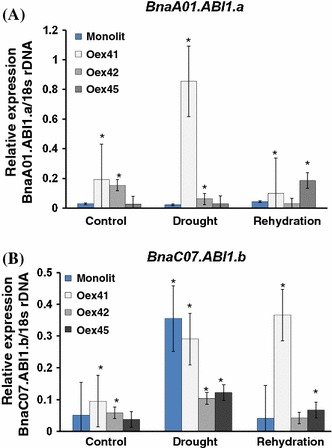
Relative expression of *BnaA01.ABI1.a* and *BnaC07.ABI1.b* genes in wild type and *ABI1*-overexpressing lines. Results were normalized against 18S rDNA expression. Data are representative of three independent experiments. Values represent mean ± SE

The plasticity of ABA signaling in response to stress in polyploids or paleopolyploids can be addressed by investigation of the paralogous *ABI1* endogenes in *B. napus*. We analysed their specificity and functional divergence by examining their involvement in the response to drought. We also asked whether *AtABI1* overexpression in the *B. napus* genetic background resulted in co-regulation of transcriptional activity of the endogenous *BnABI1* genes. The expression profiles of selected *BnaABI1* endogenes were determined using a qPCR assay in wild-type and transgenic plants under control, drought and rehydration conditions (Fig. 6).

First, to identify any functional redundancy or divergence within the *BnaABI1* paralogues, two evolutionarily distant examples described above (*BnaA01.ABI1.a* and *BnaC07.ABI1.b*), were used. Both genes showed different expression patterns during drought stress in WT and in the transgenic plants generated (Fig. 6). Under well-watered conditions, transcript levels of both *BnaA01.ABI1.a* and *BnaC07.ABI1.b* in WT plants and transgenic lines were low. However, significant changes in the induction of these genes were observed when the plants were subjected to drought stress. In general, *BnaA01.ABI1.a* expression did not change in either wild-type or transgenic plants, and remained suppressed; an exception was Oex41, which showed a significant increase in *AtABI1* (and possibly *BnaA01ABI1.a*) expression compared to the wild-type plants. In contrast, *BnaC07.AB11.b* was upregulated in both wild-type plants and all transgenic lines exposed to drought. Following rehydration, the expression of *BnaC07.ABI1.b* returned to control levels in wild-type plants and transgenic lines (Fig. 6).

### Analysis of *BnaA01.ABI1.a* and *BnaC07.ABI1.b* promoter activity

**Fig. 7:**
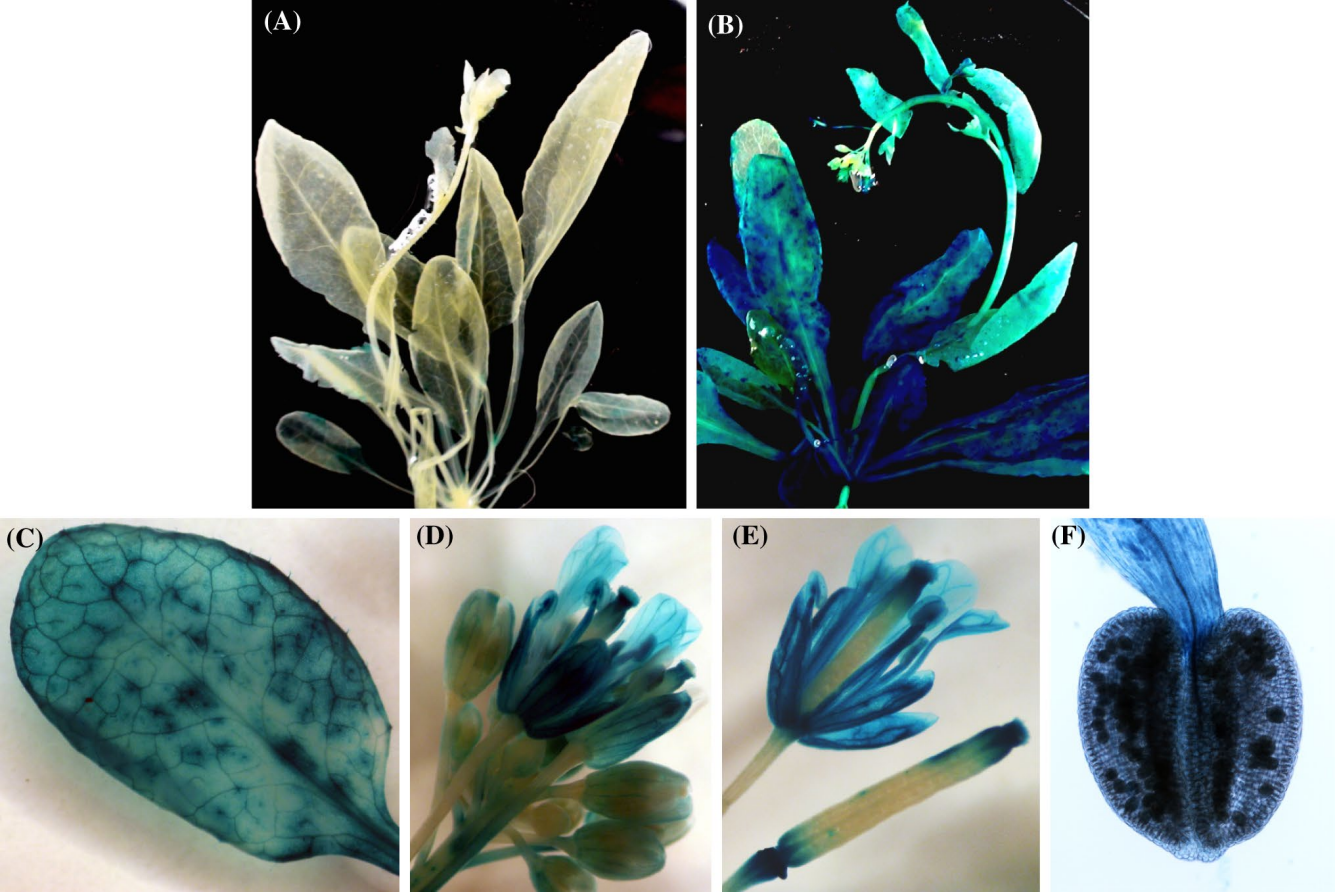
Localization of *Pro*BnaA01.ABI1.a:GUS and *Pro*BnaC07.ABI1.b:GUS activity (blue staining) in transgenic *A. thaliana* plants. A-mock, B-whole plant, C-leaves, D-flowers, E-siliques, F-pollen. Experiments were repeated (n = 3) with similar results and representative data are shown

**Fig. 8:**
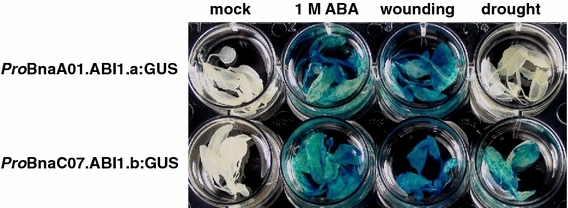
Histochemical visualization of GUS activity (blue staining) directed by *BnaA01.ABI1.a* and *BnaC07.ABI1.b* upstream regions in *A. thaliana* transgenic plants. Experiments were repeated (n = 3) with similar results and representative data are shown

**Fig. 9:**
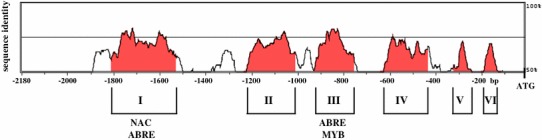
Conservation of the *BnaA01.ABI1.a* and *BnaC07.ABI1.b* promoter regions in *B*. *napus*. Six conserved regions named “Conserved Non-Coding Sequences” were defined by sequence similarity at the level of >70 % over a 90 bp interval. The sequences conserved between both promoters were visualized using VISualization Tool for Alignments (Vista) tools mVista and rVista (http://rvista.dcode.org/)

To further investigate the expression patterns of the *BnaA01.ABI1.a* and *BnaC07.ABI1.b* genes, their 2.2 kb promoter fragments were fused to the GUS reporter gene and the resulting *Pro*BnaA01.ABI1.a:GUS and *Pro*BnaC07.ABI1.b:GUS constructs were used to generate transgenic *A. thaliana* plants. Five independent transgenic lines for each construct were tested for GUS activity. All construct-specific lines showed the same expression pattern under any conditions tested. Histochemical staining of plants harboring the *Pro*BnaA01.ABI1.a:GUS and *Pro*BnaC07.ABI1.b:GUS constructs showed that there were no major differences in GUS activity in different organs (Fig. 7). Both promoters induced GUS activity in leaves, flowers, siliques and pollen (Fig. 7c–f) and in response to exogenous ABA treatment and wounding (Fig. 8). Interestingly, only *BnaC07.ABI1.b* was induced in the leaves and stems during drought (Fig. 8). This observation confirmed the earlier findings indicating different expression patterns of two *BnaABI1* paralogues under drought stress conditions (Fig. 6). To better understand transcriptional regulation of the *BnaA01.ABI1.a* and *BnaC07.ABI1.b* genes, a *de novo* search for ABA- and water deficit-responsive *cis*-regulatory elements within the 2.2 kb regions upstream of their translation start codons was performed. Based on data from different databases (PLACE, Plant Matrix Family Library at Genomatix platform and PlantCare) it was shown that the *BnaA01.ABI1.a* and *BnaC07.ABI1.b* promoters contained 20 and 14 ABA- and osmotic stress-responsive *cis*-elements within the 2.2 kb upstream of the translation start site, respectively (Table S3). They include known *cis*-acting plant elements such as ABREs, MYB recognition sites, NYC recognition sites and SALTs, which are arranged in several copies throughout the upstream regions. The sequence similarity between the *BnaA01.ABI1.a* and *BnaC07.ABI1.b* promoter regions is 56.1 % which as expected is much lower than between their protein-coding sequences (Fig. S8). Comparison of both promoter sequences allowed us to identify six conserved regions with almost 70 % sequence similarity over at least 90 bp (Figs. 9 and S8). These regions covered over 52 % of each promoter and included some common regulatory elements located at similar relative positions. Detailed comparative studies of regulatory motifs involved in abscisic acid responsiveness such as ABRE, NAC, MYB and SALT revealed variations in their numbers and genome localization in the two promoter regions (Table S3; Fig. S8). Only four common protein-binding elements were identified in the conserved regions (Fig. 9). These data imply that sequence variation in the *BnaA01.ABI1.a* and *BnaC07.ABI1.b* promoter regions, outside the conserved regions, may be responsible for changes in their induction under drought stress (Figs. 6 and 8). Especially that some of drought-responsive elements such as MYB were identified in 4 copies within 600–800 bp promoter region of drought-inducible *BnaC07.ABI1.b* paralogue; meaningfully, drought-insensitive *BnaC01.ABI1.a* paralogue misses that regulatory boxes in the corresponding promoter region.

## Discussion

In this report, we show that the role of the *ABI1* orthologous gene (unique gene copy from the *Arabidopsis* genome) is conserved in Brassicaceae. We investigated the effect of *AtABI1* overexpression in *B. napus* in drought stress conditions. Our results on the role of ABA signaling in drought stress, in particular the involvement of ABI1 protein phosphatase, highlight the complexity of the *B. napus* stress response mechanism. The transgenic *B. napus* lines overexpressing *AtABI1* were characterized in detail with respect to their drought stress response. When overexpressed in *B. napus*, *AtABI1* negatively influences several important cellular processes such as plant water content (for example, line Oex41, which has a high *ABI1* transcript level, suffered water loss of 40 %), chlorophyll accumulation and the expression profile of several ABA- and/or dehydration stress-inducible genes (Figs. 1, 2 and 5). Intriguingly, most genes with the exception of *BnaA01.ABI1.a* and *BnaX.MAPKKK18* were transcriptionally induced in control plants in a similar way to counterparts in *A. thaliana*. The expression profiles of selected PP2Cs, such as *BnaA01.ABI1.a*, *BnaC07.ABI1.b*, *BnaX.ABI2,BnaX.HAB2.a* and *BnaX.HAB2.c*, were also similar to those found in *B. oleracea* exposed to drought stress (Ludwików et al. 2013). Thus, our results highlight the regulatory functions of the ABI1 PP2C, which is highly conserved within the Brassicaceae family, and suggest that it plays a role as a regulatory hub protein (for review Ma et al. 2009) for dehydration stress responses in plants. Our results showing that the expression levels of several ABA-regulated genes were reduced in each transgenic line exposed to drought stress are consistent with such a role for ABI1 as a regulator of the drought stress response. Our previous results, which showed that ABI1 negatively regulates expression of *RAB18*, *MAPKKK18* and other drought-induced genes (Ludwików et al. 2009), supports this hypothesis.

Understanding the biological role of duplicated genes, and particularly whether they have redundant or divergent functions in stress responses, is currently a high priority, although it is a challenging task in crop plant studies. We demonstrate differential involvement of the *B. napusABI1* paralogues in the water-stress response. There are six *ABI1*-related genes in the *B. napus* genome, consistent with previous genetic studies indicating the presence of several *ABI1* gene copies in the *Brassica* genomes (Sadowski and Quiros 1998; Quiros et al. 2001; Ludwików et al. 2013). By analysis of the genome sequences of *B. rapa* and *B. oleracea* and of different sets of *B. napus* EST clones, the six copies disclosed represent the complete set of *BnaABI1* paralogues in *B. napus*. Prior to the current work, single *ABI1*-like gene had been described in the *B. napus* genome, corresponding to that identified and characterized herewith as *BnaC01.ABI1.a* (Zhang et al. 2014; Fig. S2). As expected, a phylogenetic analysis of group A PP2Cs in *A. thaliana* and *B. napus* confirmed their close evolutionary relationship (Fig. S2). Generally, the number of multiple copies of *BnaABI1* correspond proportionally to the increase in total gene number in *B. napus* polyploid genome. This suggests that a repertoire of protein targets recognized by numerous BnaABI1 *s is enlarged.* Despite the difference in the number of genes in both species, they divided into two subgroups as in other species (Xue et al. 2008; Komatsu et al. 2009).

The maintenance of several duplicated copies of key regulatory genes over long periods of evolution is consistent with the gene balance hypothesis, which assumes that elements of complex signaling networks are preferentially protected against gene loss (Blanc and Wolfe 2004; Wang et al. 2011). Certainly, if such duplicated gene copies acquire different functions, they are more likely to be stably retained in a given genome. Studies on different organisms reveal that members of gene families may respond differently to environmental conditions as a result of either sub- and/or neofunctionalization (for example, Whittle and Krochko 2009; Liu and Adams 2010). Using a custom-made *Arabidopsis* cDNA macroarray, we previously revealed differences in the gene expression profiles of PP2C (group A) members under drought conditions and after ABA treatment in *B. oleracea* and *A. thaliana* (Ludwików et al. 2013). Interestingly, the *BolABI1* gene (detected by *AtABI1*-gene-specific primers) was downregulated during drought stress in *B. oleracea*; this suggests that it is not essential for the drought stress response. On the other hand, subsequent studies indicate that one of the *BolABI1* genes in *B. oleracea* corresponding to *BnaC08.ABI1.c* in *B. napus* is induced following exogenous ABA treatment and in the early stages of drought stress, confirming its involvement in the ABA signaling pathway (Yuan et al. 2013).

To determine the degree of conservation of the transcriptional regulation of the *BnaABI1* paralogues, *BnaA01.ABI1.a* and *BnaC07.ABI1.b*, which represent the closest and a more distant homologues of the *A. thalianaAtABI1* gene, these genes were characterized in detail (Fig. S2). Both *BnaABI1* paralogues have a similar gene structure, with four exons and three introns, a C-terminal PP2C catalytic domain architecture with the same arrangement of 11 motifs, and identical localization of the NLS-like motif (Figs. S3, S5 and S7). Multiple alignments of genomic, cDNA and predicted protein sequences showed a high degree of sequence similarity between *BnaA01.ABI1.a* and *BnaC07.ABI1.b* of up to almost 85 %. However, our analysis reveals sequence variability in the 5′ upstream regions including the promoter, exon I and intron sequences, enabling gene-specific analyses at both the transcriptional and proteomic levels (Fig. S6). These data confirmed previous results indicating reduced sequence conservation of these upstream regions among members of the same gene family, which may reflect changes in the expression pattern of the respective genes (Chen et al. 2011). Our finding that the transcriptional expression patterns of selected *BnaABI1* endogenous paralogues, i.e. *BnaA01.ABI1.a* and *BnaC07.ABI1.b,* significantly differ in response to drought stress, while showing similar transcriptional patterns after treatment with other stimuli (Figs. 6, 8), is entirely novel. Both genes appeared to be responsive to exogenous ABA, but only *BnaC07.ABI1.b* is induced by drought. Thus, *BnaA01.ABI1.a* and *BnaC07.ABI1.b* endogenes may have developed new subfunctions that improve the fine-scale response to drought stress. It remains possible that further subfunctions have evolved in other *B. napusABI1* and *PP2C* group A homologues. Similarly, the different expression patterns under drought were found for two *BnaHAB2* gene copies in WT plants and *AtABI1* overexpressed lines of *B. napus*, revealing gene subfunctionalisation in the ABA signaling network (Fig. 5). This is a promising area of investigation as a comparative analysis of the *B. rapa* transcriptome (Lee et al. 2008) indicates that *B. rapa* homologues corresponding to *BnaA01.ABI1.a* and *BnaC07.ABI1.b* also differ in the response to salt stress: the *B. rapa* homologue of *BnaA01.ABI1.a* was found to be responsive to salt stress while, in contrast, a homologue of *BnaC07.ABI1.b* was not. In light of our observations, we assume that *BnaABI1* homologues are potentially drought- as well as ABA-responsive, as these two stimuli positively induces all ABI1 phosphatases studied so far in different species.

Presently, little is known about the transcriptional regulation of the PP2C gene family in plants. A previous study revealed that the *A. thalianaABI1* promoter is active in guard cells and root meristem (Leung et al. 2006). To approximate a mechanism controlling induction of both *BnaABI1* promoters under ABA treatment and drought stress, in silico identification of ABA-inducible and stress-responsive elements within these regulatory regions was performed. Previous studies on the mechanisms of transcriptional regulation of ABA-inducible genes identified specific *cis*-acting elements associated with the responses to ABA, osmotic and water stresses (Narusaka et al. 2003; Yamaguchi-Shinozaki and Shinozaki 2005, 2006). Among the best described are the ABRE (ABA-responsive element), MYB, MYC, NAC, DRE, SALT and W-box elements recognized by the *AREB*/*ABF*, *MYB*, *MYC*, *NAC*, *DREB*, *Alphin* and *WRKY* family transcription factors, respectively (Abe et al. 2003; Yamaguchi-Shinozaki and Shinozaki 2005; Wang et al. 2009). Some reports document the interaction between *ABI1* and transcription factors in *Arabidopsis* (Himmelbach et al. 2002; Cui et al. 2013). Thus, *AtMYB20*, which binds to the MYB recognition sequence (TAACTG) and the ACGT core element in the *ABI1* and *AtPP2CA* promoter regions, acts as a negative regulator of *PP2C* genes, thereby enhancing salt tolerance (Cui et al. 2013). Interestingly, sequence comparison of *AtABI1* and both *BnaABI1* promoter regions showed that these *cis*-acting elements binding AtMYB20 are only present in the *BnaA01.ABI1.a* gene (data not shown). This suggests that *AtABI1* and one, at least, of the *B. napus BnaABI1*-like genes may be involved in the same NaCl-stress-responsive pathways. An earlier study also demonstrated physical interaction between the homeodomain transcription factor AtHB6 and ABI1, which was positively correlated with PP2C activity (Himmelbach et al. 2002). To identify potentially all *cis*-acting elements associated with the response to ABA and/or drought stress within the *BnaA01.ABI1.a* and *BnaC07.ABI1.b* promoters, plant databases such as Plant Matrix Family Library, PLACE and PlantCare were used, revealing several ABA-inducible and stress-responsive elements (Table S3). Comparative sequence analysis of the *BnaA01.ABI1.a* and *BnaC07.ABI1.b* promoters showed significant differences in number and localization of ABA- and stress-inducible regulatory elements, despite the close relationship of these genes (Table S3 and Fig. S8). Interestingly, most elements are located from −1180 to −1680 bp and from −680 to −1180 bp in the *BnaA01.ABI1.a* and *BnaC07.ABI1.b* promoters, respectively, i.e. outside the most conserved regions. Only four of the regulatory motifs detected are located in the CNSs of both promoters (Fig. 9). In summary, comparison of the two promoters suggests that the lack of induction of *BnaA01.ABI1.a* by drought could result from the loss of several *cis*-elements in the *BnaA01.ABI1.a* promoter during the course of evolution. This indicates that specific expression patterns of these homologous genes, resulting in their subfunctionalization, may be modulated through mutation and possibly also through epigenetic changes within their respective promoter regions.

## Electronic supplementary material

Table S1 List of primers used in this study (DOC 49 kb)

Table S2 List of clones used to a phylogenetic analysis (DOC 35 kb)

Table S3 List of *cis*-acting elements involved in ABA and drought stress identified in *BnaA01.ABI1.a* and *BnaC07.ABI1.b* promoter regions based on PLACE (http://www.dna.affrc.go.jp/PLACE/), Plant Matrix Family Library (MathInspector, Plant IUPAC Library Version 7.0 restricted to *A. thaliana* with IUPAC search parameters: max. 0% mismatches; http://www.genomatix.de) and PlantCare (http://bioinformatics.psb.ugent.be/webtools/plantcare/html) databases (DOC 76 kb)

Figure S1 *AtABI1* transcript levels in the wild-type Monolit and *AtABI1*-overexpressing *B. napus* transgenic lines. Expression of *AtABI1* was measured by qRT-PCR (n = 3, ± SE) in leaves of control (well-watered) plants. *AtABI1* mRNA abundance was normalized against 18S rDNA expression. Error bars represent SE (PPT 79 kb)

Figure S2 Phylogenetic analysis of ABI1 proteins of *B. napus* and its diploid *B. rapa* and *B. oleracea* progenitors. ABI1 proteins of the *Brassica* species were named according to the nomenclature system proposed for the *Brassica* genus (Østergaard and King 2008). Additionally, the protein sequences derived from JX122895 (Zhang et al. 2014) and KF577723 (Yuan et al. 2013) clones were included. The root tree was constructed using ClustalX to generate alignments of the studied amino acid sequences and selected fragments corresponding to the PP2C catalytic domain. Evolutionary analyses were conducted in MEGA5.1. The percentage of replicate trees is shown on the branches and is calculated according to the bootstrap test (1000 replicates) (PDF 13 kb)

Figure S3 Multiple sequence alignment of full-length ABI1 proteins from *A. thaliana* and *B. napus*. Sequences were aligned using the ClustalX program with default parameters. Gaps for optimal alignment are indicated by dashes. Asterisks beneath sequences indicate identical amino acid residues. The gray background and red/blue letters mark the catalytic domain and 11 characteristic motifs, respectively, as assigned by Bork et al. (1996), which are highly conserved across the *ABI1* gene family in *A. thaliana* and *B. napus*. The amino acid position is given on the right of each sequence. The NLS-like (monopartite nuclear localization signal) motif is underlined (DOC 24 kb)

Figure S4 Alignments of (A) *BnaA01.ABI1.a* (*B. napus*), *BraA01.ABI1.a* (*B. rapa*) and *BolC01.ABI1.a* (*B. oleracea*) genomic DNA sequences; (B) *BnaC07.ABI1.b* (*B. napus*), *BraA03.ABI1.b* (*B. rapa*) and *BolC07.ABI1.b* (*B. oleracea*) genomic DNA sequences. These sequences were obtained using the same *BnaABI1* gene-specific primer pair. Introns are shown in gray. The names of *B. napus* and *B. rapa*, and *B. napus* and *B. oleracea*, orthologous sequences are marked in red. Asterisks indicate identical residues (DOC 33 kb)

Figure S5 Schematic diagram representing the structure of the *BnaA01.ABI1.a* and *BnaC07.ABI1.b* genes in *B. napus*. The exons are shown as thick yellow boxes, the introns as lines and the 3’ and 5’ UTRs as thick blue boxes. The exon/intron structure of each *BnaABI1* gene was defined by comparison of their genomic and cDNA sequences. The 5′ and 3’ end of *BnaABI1* genes was predicted by five independent 5′ and 3’ RACE reactions. The scale is shown below in base pairs. The gene structure of both *BnaABI1* genes was illustrated using the Gene Structure Display Server (http://gsds.cbi.pku.edu.cn/) (PNG 8 kb)

Figure S6 Sequence alignment of *BnaA01.ABI1.a* and *BnaC07.ABI1.b* fragment genomic DNA including exon I and intron I. Introns are shown in gray. Asterisks indicate identical residues. Translation initiation codons (ATG) are shown (DOC 24 kb)

Figure S7 Localization of the conserved PP2C catalytic domain in deduced BnaABI1 protein sequences. The PP2C domain family was defined and mapped in BnaABI1 protein sequences using The Conserved Domain Database of the NCBI (http://www.ncbi.nlm.nih.gov/Structure/cdd/wrpsb.cgi). The scale is shown at the top in number of amino acids (TIFF 37 kb)

Figure S8 Alignment of *BnaA01.ABI1.a* and *BnaC07.ABI1.b* promoter regions. The six CNS (Conserved Non-Coding Sequence) regions were identified using VISualization Tool for Alignments (Vista) tools mVista and rVista (http://rvista.dcode.org/) with sequence similarity at the level of >70% over a 90 bp region; they are highlighted in red. The putative regulatory elements identified by public database searches are boxed in green and named above the core sequence. Gaps for optimal alignment are indicated by dashes. Asterisks beneath sequences indicate identical nucleotide residues (DOC 48 kb)
