# Supplementary material for: Involvement of genes encoding ABI1 protein phosphatases in the response of Brassica napus L. to drought stress
Source: Plant Mol Biol. 2015 Jun 10;88(4-5):445–57. doi: 10.1007/s11103-015-0334-x (PMC4486095; doi:10.1007/s11103-015-0334-x)
Supplement: Supplementary file 1 — List of primers used in this study (DOC 49 kb) [file 11103_2015_334_MOESM1_ESM.doc]

Article title: Involvement of genes encoding ABI1 protein phosphatases in the response of *Brassica napus* L. to drought stress

Journal name: Plant Molecular Biology

Author name: Danuta Babula-Skowrońska, Agnieszka Ludwików, Agata Cieśla, Anna Olejnik, Teresa Cegielska-Taras, Iwona Bartkowiak-Broda, Jan Sadowski

Corresponding authors: Danuta Babula-Skowrońska, Institute of Plant Genetics, Polish Academy of Sciences, Strzeszyńska 34, 60-479 Poznań, Poland; e-mail: dbab@igr.poznan.pl;

Jan Sadowski, Department of Biotechnology, Institute of Molecular Biology and Biotechnology, Faculty of Biology, Adam Mickiewicz University, Umultowska 89, 61-614 Poznań, Poland; e-mail: jsad@amu.edu.pl

Supplementary Table S1. List of primers used in this study

| Gene name | Forward primer | Sequence (5'-> 3') | Reverse primer | Sequence (5'-> 3') |
| --- | --- | --- | --- | --- |
| **Gene cloning** | | | | |
|  |  |  |  |  |
| *BnaA01.ABI1.a* | BngABI1aF | CATCATCATCATCGTCTTCGTCGTC | cABI1aR | ACAAAGGGCTTTTAGGACGTTAC |
| *BnaC01.ABI1.a* | C01ABI1aF | GCCAGTACTCGTCTCAAGATTT | cABI1aR | ACAAAGGGCTTTTAGGACGTTAC |
| *BnaA03.ABI1.b* | A03ABI1bF | TCATCGTCGTCGTCAGACAAAGTCG | A03ABI1bR | tgtggatgtggatctctcgtc |
| *BnaC07.ABI1.b* | BngABI1bF | CCCCGAGACCCCCCAGAT | BngABI1bR | AACAAGGACCATCTGCTTC |
| *BnaA08.ABI1.c* | A08ABI1cF | CACAATCAATCTCCTCTG | A08ABI1cR | GGATCTTCTCCTTCACCTCT |
| *BnaC08.ABI1.c* | C0XABI1cF | ACCAATCAATCTCCTCTG | C0XABI1cR | CTTTTCTCTCCTCCGTGA |
|  |  |  |  |  |
| **Genome/Chromosomal localization** | | | | |
| *BnaA01.ABI1.a* | cABI1aF3 | GTTGGAGAAGAGATCAACGGCT | cABI1aR3 | TCAATCCTCGCCGCTTCATCTTCT |
| *BnaC01.ABI1.a* | cABI1aaF | TCGCTGGAGAAGAGATCAAT | cABI1aR3 | TCAATCCTCGCCGCTTCATCTTCT |
| *BnaA03.ABI1.b* | cABI1bF | GTTTCTCCGGCGGTTGCTAT | cABI1bR | CTCTCTACAATAGTCCGCTAC |
| *BnaC07.ABI1.b* | cABI1bbF | GATTCCGACAACAACGGCGAGA | cABI1bbR | AGCGTCGGATTCTCCCTGGCCA |
| *BnaA08.ABI1.c* | cABI1cF | CATCATCAGATATAGCCGCAG | cABI1cR | GCAATAGTTCGCTACCTACAC |
| *BnaC08.ABI1.c* | cABI1ccF | AATCATCACCCGCTGATGCTG | cABI1cR | GCAATAGTTCGCTACCTACAC |
|  |  |  |  |  |
| **Promoter isolation** | | | | |
|  |  |  |  |  |
| *BnaA01.ABI1.a* |  |  | nABI1a51 | TCTCCGGTCTCCTCCCACAGATCGAAG |
|  |  |  | nABI1a52 | AAGTTAGGTGAGTTGATCCGCGAGGTC |
| *BnaC07.ABI1.b* |  |  | nABI1b51 | TTCTCCGGATGATGATGATGCCGAAAC |
|  |  |  | nABI1b52 | TCTCGCCGTTGTTGTCGGAATCTTGAG |
| **Vector construction** | | | | |
|  |  |  |  |  |
| *BnaA01.ABI1.a* | DanBnABI1aF | CACCACTTCGTCCCTTAATCGCGT | DanBnABI1aR | ACGGCTGGTGATACTTCCTCCATT |
| *BnaC07.ABI1.b* | DanBnABI1bF | GAACCTCCTTAGTCCTTGCTTC | DanBnABI1bR | TCTCGCCGTTGTTGTCGGAAT |
| *AtABI1* | ABI1FBamHI | _GGATCC_ATGGAGGAAGTATCTCC | ABI1RXo | _GCCTCGAG_TCAGTTCAAGGGTTTGCTC |
| NOS | NOStF1 | CGAACACTTGATACATGTGCCT | BARR1 | GAAGTTGACCGTGCTTGTCTC |
|  |  |  |  |  |
| **qPCR analysis** | | | | |
|  |  |  |  |  |
| *AtABI1* | AtABI1F | AGTGGAATGGAGCTCGTGTT | AtABI1R | ACTTCCGGATCAGGAATGATCGAT |
| *BnaA01.ABI1.a* | BnABI1aF | GGTGGAACGGAGCTCGTGTG | BnABI1aR | CTTCCGGATCAGGAATGATCGAT |
| *BnaC07.ABI1.b* | BnABI1bF | GGTGGAACGGAGCTCGTGTC | BnABI1bR | CTTCCGGATCGGGAATGATCGAC |
| *BnaX.ABI2* | AtABI2F | TGAAGCGGCGAGGATAGAAG | AtABI2R | ATACACGAGCCCCGTTCCA |
| *BnaX.HAB2.a* | Bo1HB2F | TGTGATACCAGAGCCCGAAGTGAC | Bo1HB2R | CCTACACCTCTCTCAGCTAAAGGC |
| *BnaX.HAB2.c* | Bo3HB2F | CCACGGAGGCCTACACGTTGC | Bo3HB2R | CCCACTGCACCTGAAGCCTCC |
| *BnaX.MAPKKK1* | MEK1-70F | TTGTTATATTCATCATGAGAGGCGAATC | MEK1-70R | GCTTGTCAAGATCTTGCTGACACCA |
| *BnaX.MAPKKK18* | qMK18Fb | TGCGCATTGCGACATTAAGG | qMK18Rb | AACCCACCGCCCATATATCACT |
| *BnaX.ERF1* | ERF1F | CACCGCTCCGTGAAGTTAGATA;  Ludwików et al. 2009 | ERF1R | ACCCCAAAAGCTCCTCAAGGTA; Ludwików et al. 2009 |
| *BnaX.RD19A* | RD19aF | GCGACTCTGGTTGCAATGG;  Ludwików et al. 2009 | RD19aR | TCATGAGCCCTCCGGTTTT;  Ludwików et al., 2009 |
| *BnaX.RAB18* | *RAB18F* | GGAGAAGTTGCCAGGTCATCATG; Ludwików et al. 2009 | *RAB18R* | CACCGTAGCCACCAGCATCATA  Ludwików et al. 2009 |
| *18S rDNA* | 18SF | GGTCTGTGATGCCCTTAGATGTT | 18SR | GGCAAGGTGTGAACTCGTTGA |
|  | | | | |
| **5' and 3' RACE** | | | | |
|  |  | 5' RACE |  | 3' RACE |
|  |  |  |  |  |
| *BnaA01.ABI1.a* | rA01ABI1a5 | CAACAACTATCTCCGACGACGACGAA | rA01ABI1a3 | CGATGTCCGCGGCTGAGTACTT |
| *BnaC07.ABI1.b* | rC07ABI1b5 | GGATGATGATGATGATGCCGAAAC | rC07ABI1b3 | TGCTCACGGAGGAGAGAAAAGG |
