# Supplementary material for: Involvement of genes encoding ABI1 protein phosphatases in the response of Brassica napus L. to drought stress
Source: Plant Mol Biol. 2015 Jun 10;88(4-5):445–57. doi: 10.1007/s11103-015-0334-x (PMC4486095; doi:10.1007/s11103-015-0334-x)
Supplement: Supplementary file 2 — List of clones used to a phylogenetic analysis (DOC 35 kb) [file 11103_2015_334_MOESM2_ESM.doc]

Article title: Involvement of genes encoding ABI1 protein phosphatases in the response of *Brassica napus* L. to drought stress

Journal name: Plant Molecular Biology

Author name: Danuta Babula-Skowrońska, Agnieszka Ludwików, Agata Cieśla, Anna Olejnik, Teresa Cegielska-Taras, Iwona Bartkowiak-Broda, Jan Sadowski

Corresponding authors: Danuta Babula-Skowrońska, Institute of Plant Genetics, Polish Academy of Sciences, Strzeszyńska 34, 60-479 Poznań, Poland; e-mail: dbab@igr.poznan.pl;

Jan Sadowski, Department of Biotechnology, Institute of Molecular Biology and Biotechnology, Faculty of Biology, Adam Mickiewicz University, Umultowska 89, 61-614 Poznań, Poland; e-mail: jsad@amu.edu.pl

Supplementary Table S2. List of clones used to a phylogenetic analysis

| *A. thaliana* | | *Brassica* |
| --- | --- | --- |
| Gene model | Gene name | Clone ID |
| At5g51760 | AHG1 | GSBRNA2T00066720001 |
|  |  | GSBRNA2T00052649001 |
| At3g11410 | AHG3 | GSBRNA2T00000310001 |
|  |  | GSBRNA2T00111040001 |
| At5g59220 | HAI1 | GSBRNA2T00053231001 |
|  |  | GSBRNA2T00133315001 |
| At1g07430 | HAI2 | GSBRNA2T00122885001 |
|  |  | GSBRNA2T00122177001 |
|  |  | GSBRNA2T00032103001 |
| At2g29380 | HAI3 | GSBRNA2T00082049001 |
|  |  | GSBRNA2T00069317001 |
| At1g17550 | HAB2 | GSBRNA2T00034904001 |
|  |  | GSBRNA2T00102986001 |
|  |  | GSBRNA2T00089279001 |
|  |  | GSBRNA2T00139675001 |
| At1g72770 | HAB1 | GSBRNA2T00102599001 |
|  |  | GSBRNA2T00103369001 |
| At4g26080 | ABI1 | Bra013945 |
|  |  | Bra019121 |
|  |  | Bra010441 |
|  |  | Bol039593 |
|  |  | Bol042273 |
|  |  | Bol029966 |
| At5g57050 | ABI2 | GSBRNA2T00065613001 |
