# Supplementary material for: Involvement of genes encoding ABI1 protein phosphatases in the response of Brassica napus L. to drought stress
Source: Plant Mol Biol. 2015 Jun 10;88(4-5):445–57. doi: 10.1007/s11103-015-0334-x (PMC4486095; doi:10.1007/s11103-015-0334-x)
Supplement: Supplementary file 3 — List of cis-acting elements involved in ABA and drought stress identified in BnaA01.ABI1.a and BnaC07.ABI1.b promoter regions based on PLACE (http://www.dna.affrc.go.jp/PLACE/), Plant Matrix Family Library (MathInspector, Plant IUPAC Library Version 7.0 restricted to A. thaliana with IUPAC search parameters: max. 0% mismatches; http://www.genomatix.de) and PlantCare (http://bioinformatics.psb.ugent.be/webtools/plantcare/html) databases (DOC 76 kb) [file 11103_2015_334_MOESM3_ESM.doc]

Article title: Involvement of genes encoding ABI1 protein phosphatases in the response of *Brassica napus* L. to drought stress

Journal name: Plant Molecular Biology

Author name: Danuta Babula-Skowrońska, Agnieszka Ludwików, Agata Cieśla, Anna Olejnik, Teresa Cegielska-Taras, Iwona Bartkowiak-Broda, Jan Sadowski

Corresponding authors: Danuta Babula-Skowrońska, Institute of Plant Genetics, Polish Academy of Sciences, Strzeszyńska 34, 60-479 Poznań, Poland; e-mail: dbab@igr.poznan.pl;

Jan Sadowski, Department of Biotechnology, Institute of Molecular Biology and Biotechnology, Faculty of Biology, Adam Mickiewicz University, Umultowska 89, 61-614 Poznań, Poland; e-mail: jsad@amu.edu.pl

| ***BnaA01.ABI1.a*** | | | | | | | | | | | |
| --- | --- | --- | --- | --- | --- | --- | --- | --- | --- | --- | --- |
|  |  | | Cis-acting element, TF |  |  | Strand | Sequence | Function | PLACE | Plant Matrix Family Library | PlantCare |
| P$FAM266 | P$MYB1AT | | MYB | 68 | 73 | + | **TAACCA** | ABA; MYB recognition site found in the promoters of the dehydration-responsive gene | **+** |  |  |
| P$MYCL | P$ICE.01; | | ICE (inducer of CBF expression 1), AtMYC2 (rd22BP1) Myc-like basic helix-loop-helix binding factors | 124 | 142 | - | gaaatca**ACAT**atgcttaa | AtMYC2 is common transcription factor of light, ABA and JA signaling pathways |  | **+** |  |
| P$FAM263 | P$DPBFCOREDCDC3 | | "DPBF-1 and 2 (Dc3  promoter-binding factor-1 and 2); ABI5" | 308 | 314 | + | **ACACAAG** | Induced by ABA | **+** |  |  |
| P$NACF | P$ANAC019.01 | | ANAC019; Arabidopsis NAC domain containing protein 19 | 312 | 338 | + | aagtggcataaaaca**TACG**gaacttgg | Induced by drought, high salinity and abscisic acid (ABA). Slightly up-regulated by jasmonic acid. Not induced by cold treatment |  | **+** |  |
| P$NACF | P$ANAC019.01 | | ANAC019; Arabidopsis NAC domain containing protein 19 | 456 | 482 | + | gaaagacaagatatt**TACG**gaattaaa | Induced by drought, high salinity and abscisic acid (ABA). Slightly up-regulated by jasmonic acid. Not induced by cold treatment |  | **+** |  |
| P$SALT | P$ALFIN1.01 | | Zinc-finger protein in alfalfa roots, regulates salt tolerance | 530 | 544 | - | gttgg**GTGG**cgggag | Salt/drought responsive elements |  | **+** |  |
| P$FAM013 | P$LTRECOREATCOR15 | | COR15, BN115 (B. napus) | 554 | 560 | + | **TCCGACC** | Core of low temperature responsive element; ABA responsiveness; drought; Involved in cold induction of BN115 gene from winter *Brassica napus* | **+** |  |  |
| ABRE |  | | ABRE | 556 | 562 | - | **TACGGTC** | Involved in the abscisic acid responsiveness |  |  |  |
| P$FAM002 | P$ABRELATERD | | ABRE; ERD1 | 560 | 572 | - | **GGTGACGTGTACG** | Induced by dehydration stress | **+** |  |  |
| P$SALT | P$ALFIN1.02 | | Zinc-finger protein in alfalfa roots, regulates salt tolerance | 565 | 579 | - | gagaggg**GGTG**acgt | Salt/drought responsive elements |  | **+** |  |
| P$FAM116 | P$DRE1COREZMR | | "DRE1"; rab17 | 576 | 582 | - | **ACCGAGA** | ABA; drought response | **+** |  |  |
| P$FAM002;  ABRE | P$ABREMOTIFAOSOSEM | | ABRE | 605 | 617 | + | **CTATACGTGTCCC** | Involved in the abscisic acid responsiveness for regulation by ABA | **+** | **+** |  |
| P$FAM013 | P$LTRECOREATCOR15 | | LTRE; COR15, BN115 (*B. napus*) | 764 | 770 | - | **TCCGACT** | Core of low temperature responsive element; ABA responsiveness; drought; Involved in cold induction of BN115 gene from winter *Brassica napus* | **+** |  |  |
| P$FAM002;  ABRE | P$ABRELATERD | | ABRE | 841 | 853 | - | **ATCTACGTGCATT** | Involved in the abscisic acid responsiveness | **+** | **+** |  |
| P$NACF | P$ANAC092.01 | | ANAC019; Arabidopsis NAC domain containing protein 19 | 860 | 886 | + | ttgcaattataaattta**CACG**aaatac | Induced by drought, high salinity and abscisic acid (ABA). Slightly up-regulated by jasmonic acid. Not induced by cold treatment |  | + |  |
| P$FAM266 | P$MYB1AT | | MYB | 942 | 947 | + | **TAACCA** | ABA; MYB recognition site found in the promoters of the dehydration-responsive gene | + |  |  |
| P$FAM002;  ABRE | P$ABREMOTIFAOSOSEM | | ABRE | 1337 | 1349 | - | **ACATACGTGTCAC** | Involved in the abscisic acid responsiveness | + | + |  |
| P$MYBL | P$MYB96.01 | | Myb domain protein 96 (MYBCOV1) | 1399 | 1415 | - | aaacttt**AGTT**aggtca | MYB-like R2R3 type Myb transcription factor whose expression is strongly induced by abscisic acid. Mediates abscisic acid signaling during drought stress response proteins |  |  |  |
| P$MYCL | P$ICE.01 | | ICE (inducer of CBF expression 1), AtMYC2 (rd22BP1) Myc-like basic helix-loop-helix binding factors | 1580 | 1598 | - | gaaaaag**ACAC**ctgctttc | AtMYC2 is common transcription factor of light, ABA and JA signaling pathways |  | **+** |  |
| P$FAM266 | P$MYB1AT | |  | 2088 | 2093 | + | **AAACCA** | ABA; MYB recognition site found in the promoters of the dehydration-responsive gene | **+** |  |  |
| ***BnaC07.ABI1.b*** | | | | | | | | | | | |
|  |  | Cis-acting element, TF | |  |  | Strand | Sequence | Function | PLACE | Plant Matrix Family Library | PlantCare |
| P$FAM059 | P$ACGTTBOX | T-box; ACGT element | | 75 | 80 | + | **AACGTT** | ABA response | **+** |  |  |
| P$FAM059 | P$ACGTTBOX | T-box; ACGT element | | 75 | 80 | - | **AACGTT** | ABA response | **+** |  |  |
| P$NACF | P$ANAC019.01; ANAC019 | Arabidopsis NAC domain containing protein 19 | | 152 | 178 | + | taaagacaagatatt**TACG**gaatttaa | Induced by drought, high salinity and abscisic acid (ABA). Slightly up-regulated by jasmonic acid. Not induced by cold treatmen |  | **+** |  |
| P$FAM002 | P$ABRELATERD | ABRE | | 236 | 248 | - | TGT**GACGTGTA**CG | Induced by dehydration stress | **+** |  |  |
| P$FAM266 | P$MYB1AT | MYB | | 985 | 990 | - | **AAACCA** | ABA; MYB recognition site found in the promoters of the dehydration-responsive gene | **+** |  |  |
| P$NACF | P$ANAC019.01 | Arabidopsis NAC domain containing protein 19 | | 1019 | 1045 | - | atttaatccgaaata**CACG**aaagttta | Induced by drought, high salinity and abscisic acid (ABA). Slightly up-regulated by jasmonic acid. Not induced by cold treatment |  | **+** |  |
| P$NACF | P$ANAC092.01; ANAC06 | Arabidopsis NAC domain containing protein 92 (ATNAC2/ATNAC6); | | 1021 | 1047 | - | agatttaatccgaaata**CACG**aaagtt | Response to oxidative stress |  | **+** |  |
| P$FAM002 | P$ABREMOTIFAOSOSEM | "motif A" ABRE-like sequence | | 1098 | 1110 | - | ACG**TACGTGTC**AC | *Cis*-acting element involved in the abscisic acid responsiveness | **+** | **+** |  |
| P$MYBL | P$MYB96.01 | Myb domain protein 96 (MYBCOV1); Encodes a R2R3 type Myb transcription | | 1167 | 1183 | - | taacttt**AGTT**aagcat | R2R3 type Myb transcription factor whose expression is strongly induced by abscisic acid. Mediates abscisic acid signaling during drought stress response |  | **+** |  |
| P$FAM266 | P$MYB1AT | MYB | | 1314 | 1319 | - | **AAACCA** | Involved in ABA responsiveness; Early responsive to dehydration | **+** |  |  |
| P$FAM266 | P$MYB1AT | MYB | | 1348 | 1353 | + | **AAACCA** | ABA; MYB recognition site found in the promoters of the dehydration-responsive gene | **+** |  |  |
| P$FAM169;  P$FAM266 | P$MYBATRD2 P$MYB1AT | MYB | | 1374 | 1380 | - | **CTAACCA** | In dehydration-responsive; ABA-induction; water stress | **+** |  |  |
| P$FAM266 | P$MYB1AT | MYB | | 1485 | 1490 | - | **AAACCA** | ABA; MYB recognition site found in the promoters of the dehydration-responsive gene | **+** |  |  |
| P$NACF | P$ANAC019.01 | *Arabidopsis* NAC domain containing protein 19 | | 1714 | 1740 | - | ggaggaggatatagg**TACG**caaaggca | Induced by drought, high salinity and abscisic acid (ABA). Slightly up-regulated by jasmonic acid. Not induced by cold treatment |  | **+** |  |
