# Supplementary figures and images for: Involvement of genes encoding ABI1 protein phosphatases in the response of Brassica napus L. to drought stress
Source: Plant Mol Biol. 2015 Jun 10;88(4-5):445–57. doi: 10.1007/s11103-015-0334-x (PMC4486095; doi:10.1007/s11103-015-0334-x)

## Slide 1
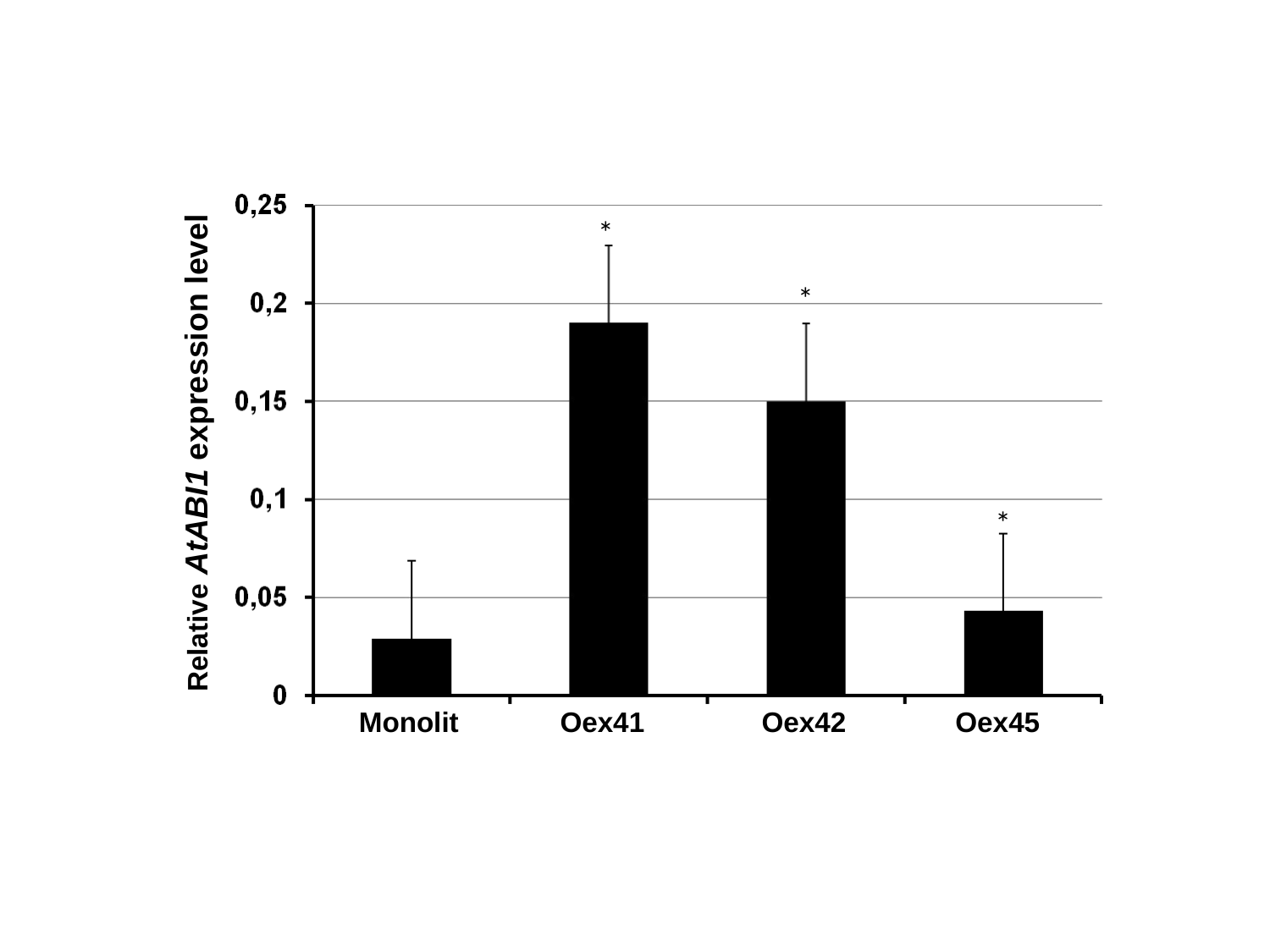

*
*
Relative AtABI1 expression level
 *
 Monolit Oex41 Oex42 Oex45

Supplement: Supplementary file 4 — AtABI1 transcript levels in the wild-type Monolit and AtABI1-overexpressing B. napus transgenic lines. Expression of AtABI1 was measured by qRT-PCR (n = 3, ± SE) in leaves of control (well-watered) plants. AtABI1 mRNA abundance was normalized against 18S rDNA expression. Error bars represent SE (PPT 79 kb) [file 11103_2015_334_MOESM4_ESM.ppt]

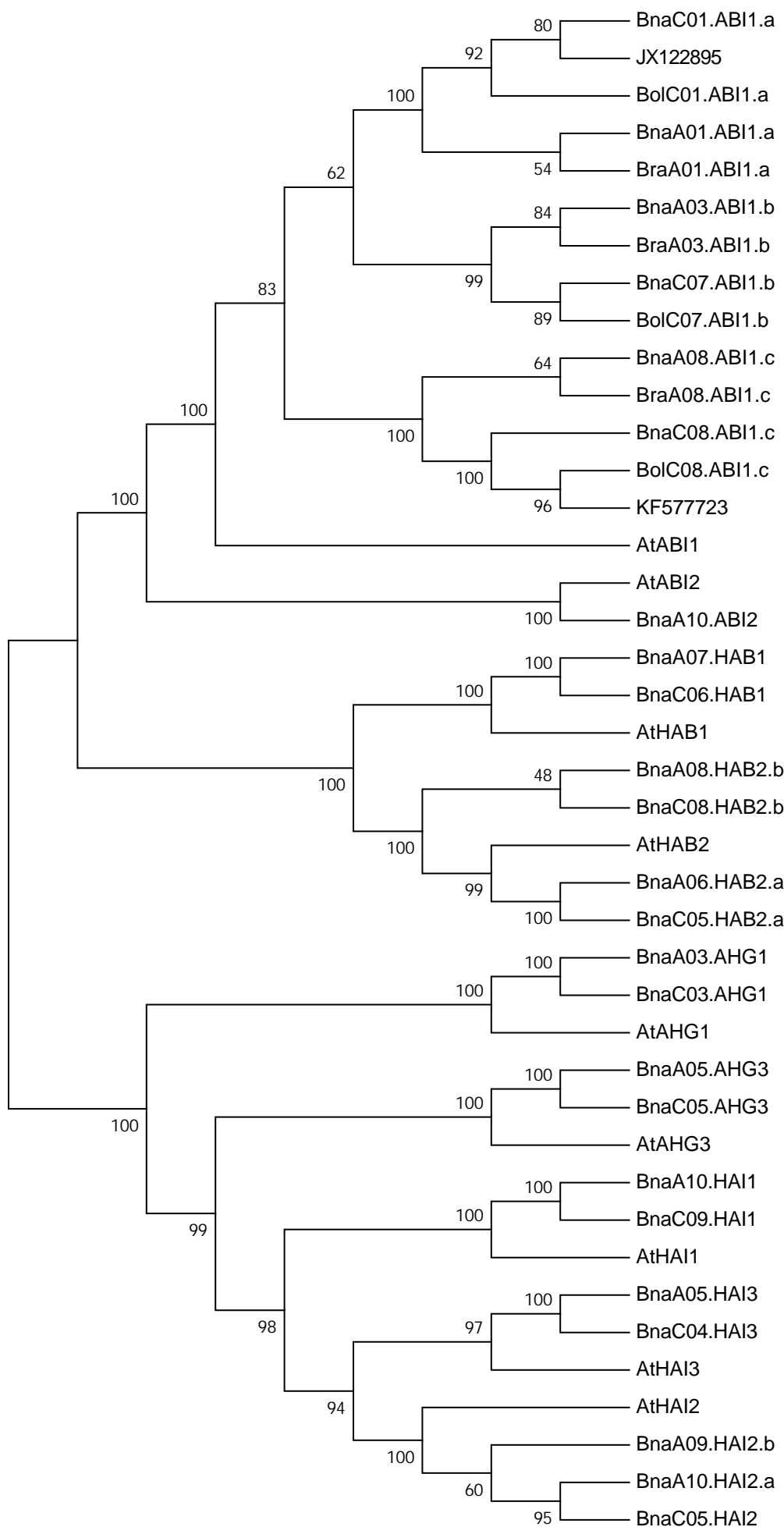

Supplement: Supplementary file 5 — Phylogenetic analysis of ABI1 proteins of B. napus and its diploid B. rapa and B. oleracea progenitors. ABI1 proteins of the Brassica species were named according to the nomenclature system proposed for the Brassica genus (Østergaard and King 2008). Additionally, the protein sequences derived from JX122895 (Zhang et al. 2014) and KF577723 (Yuan et al. 2013) clones were included. The root tree was constructed using ClustalX to generate alignments of the studied amino acid sequences and selected fragments corresponding to the PP2C catalytic domain. Evolutionary analyses were conducted in MEGA5.1. The percentage of replicate trees is shown on the branches and is calculated according to the bootstrap test (1000 replicates) (PDF 13 kb) [file 11103_2015_334_MOESM5_ESM.pdf]

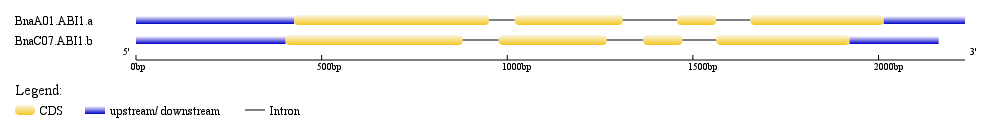

Supplement: Supplementary file 8 — Schematic diagram representing the structure of the BnaA01.ABI1.a and BnaC07.ABI1.b genes in B. napus. The exons are shown as thick yellow boxes, the introns as lines and the 3’ and 5’ UTRs as thick blue boxes. The exon/intron structure of each BnaABI1 gene was defined by comparison of their genomic and cDNA sequences. The 5′ and 3’ end of BnaABI1 genes was predicted by five independent 5′ and 3’ RACE reactions. The scale is shown below in base pairs. The gene structure of both BnaABI1 genes was illustrated using the Gene Structure Display Server (http://gsds.cbi.pku.edu.cn/) (PNG 8 kb) [file 11103_2015_334_MOESM8_ESM.png]

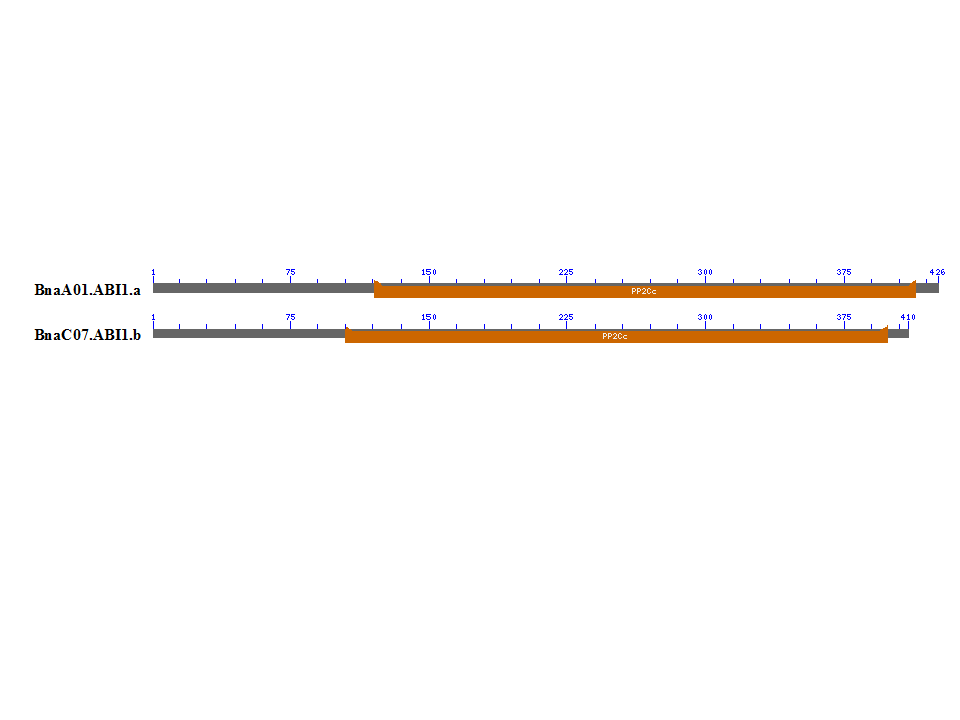

Supplement: Supplementary file 10 — Localization of the conserved PP2C catalytic domain in deduced BnaABI1 protein sequences. The PP2C domain family was defined and mapped in BnaABI1 protein sequences using The Conserved Domain Database of the NCBI (http://www.ncbi.nlm.nih.gov/Structure/cdd/wrpsb.cgi). The scale is shown at the top in number of amino acids (TIFF 37 kb) [file 11103_2015_334_MOESM10_ESM.tif]
