# Supplementary material for: Involvement of genes encoding ABI1 protein phosphatases in the response of Brassica napus L. to drought stress
Source: Plant Mol Biol. 2015 Jun 10;88(4-5):445–57. doi: 10.1007/s11103-015-0334-x (PMC4486095; doi:10.1007/s11103-015-0334-x)
Supplement: Supplementary file 6 — Multiple sequence alignment of full-length ABI1 proteins from A. thaliana and B. napus. Sequences were aligned using the ClustalX program with default parameters. Gaps for optimal alignment are indicated by dashes. Asterisks beneath sequences indicate identical amino acid residues. The gray background and red/blue letters mark the catalytic domain and 11 characteristic motifs, respectively, as assigned by Bork et al. (1996), which are highly conserved across the ABI1 gene family in A. thaliana and B. napus. The amino acid position is given on the right of each sequence. The NLS-like (monopartite nuclear localization signal) motif is underlined (DOC 24 kb) [file 11103_2015_334_MOESM6_ESM.doc]

Article title: Involvement of genes encoding ABI1 protein phosphatases in the response of *Brassica napus* L. to drought stress

Journal name: Plant Molecular Biology

Author name: Danuta Babula-Skowrońska, Agnieszka Ludwików, Agata Cieśla, Anna Olejnik, Teresa Cegielska-Taras, Iwona Bartkowiak-Broda, Jan Sadowski

Corresponding authors: Danuta Babula-Skowrońska, Institute of Plant Genetics, Polish Academy of Sciences, Strzeszyńska 34, 60-479 Poznań, Poland; e-mail: dbab@igr.poznan.pl;

Jan Sadowski, Department of Biotechnology, Institute of Molecular Biology and Biotechnology, Faculty of Biology, Adam Mickiewicz University, Umultowska 89, 61-614 Poznań, Poland; e-mail: jsad@amu.edu.pl

AtABI1 MEEVSPAIAGPFRPFSETQ-MDFTGIRLGKGYCNNQYSNQDSENGDLMVSLPETSSCSVS 59

BnaA01.ABI1.a MEEVSPAVAMPFMPFPEQQ-MELAGIMLGKGYCNGQYSSQDSENGS-------CSVSGSR 52

BnaC07.ABI1.b MEEASPAVAMPFMPFPETPQMELAGIMLGKGYCNGQYSAQDSDNNGE------TSSCSVS 54

*** *** * ** ** * * ** ******* *** *** * *

AtABI1 GSHGSESRKVLISRINSPNLNMKESAAADIVVVDISAGDEINGSDITSEKKMISRTESRS 119

BnaA01.ABI1.a KVLTSRINSPNLNMKKEPSSSSSSSSSSS-SSSSEIVVGEEINGSDERSKKMISRTESRS 111

BnaC07.ABI1.b GAQ---------SRIVSASSSSSGEGING-SDER---------STVQSEKKMISRTESRS 95

***********

motif 1 motif 2

AtABI1 LFEFKSVPLYGFTSICGR**RPEMEDAVSTIPRFL**QSSSGSMLDGRFDPQSA**AHFFGVYDGH** 179

BnaA01.ABI1.a LFEFKSVPLYXVTSICGR**RPEMEDAVSTIPRFL**QSPTNSMLDGRFNPQTT**AHFFGVYDGH** 171

BnaC07.ABI1.b LFEFKSVPLYGFTSICGR**RPEMEDAVSAIPRFL**QSPTNSLVDGRFNPQST**AHFFGVYDGH** 155

********** *************** ******* * **** ** **********

motif 3

AtABI1 **GGSQVANYCRERMHLALAE**EIAKEKPMLCDGDTWLEKWKKALFNSFLRVDSEIESVAPET 239

BnaA01.ABI1.a **GGSQVANYCRERMHLALAE**EIAKEKPMLCDGDTWQEKWKKALFNSFLRVDSEVESVAPET 231

BnaC07.ABI1.b **GGSQVAEYCRERMHLALAE**EMARENPTLCDGDTWQEKWKRALFNSFLRVDLEIESVAPET 215

****** ************* * * * ******* **** ********** * *******

AtABI1 **VGSTSVVAVVFPSHIFVANCGDSRAVLCR**GKTALPLSVDHKPDREDEAARIEAAGGKVIQ 299

BnaA01.ABI1.a **VGSTSVVAVVFPTHIFVANCGDSRAVLCR**GKTALPLSTDHKPDREDEAARIEAAGGKVIR 291

BnaC07.ABI1.b **VGSTSVVAVVFPTHIFVANCGDSRAVICR**GKTALPLSTDHKPDREDEAERIEAAGGKVIR 275

************ ************* ********** ********** **********

motif 6 motif 7 motif 8 motif 9

AtABI1 WNG**ARVFGVLAMSRSIGDRYLKP**SIIP**DPEVTAVKRVKEDDCLILASDGVWDVMTDEEAC** 359

BnaA01.ABI1.a WNG**ARVFGVLAMSRSIGDRYLKP**SIIP**DPEVTAVRRVKEDDCLILASDGVWDVMTDEEAC** 351

BnaC07.ABI1.b WNG**ARVFGVLAMSRSIGDRYLKP**SIIP**DPEVTAVRRVKEDDCLILASDGVWDVMTDEEAC** 335

********************************** *************************

motif 10 motif 11

AtABI1 **EMARKRIL**LWHKKNAVAGDASLLADERRKEGKDPA**AMSAAEYLSKLAIQRGSKDNISVVV** 419

BnaA01.ABI1.a **EMARKRIL**LWHKKNAVAGDASLHTDERRGEGKDPA**AMSAAEYLSKLALQRGSKDNITVVV** 411

BnaC07.ABI1.b **EMARKRIL**LWHKKNAMAGDASLLTNERRGEGKDPA**AMSAAEYLSKLALQRGSKDNITVVV** 395

*************** ****** *** ****************** ******** ***

AtABI1 **VDLK**PRRKLKSKPLN 434

BnaA01.ABI1.a **VDLK**PQRKFKSKPLN 426

BnaC07.ABI1.b **VDLK**PQRKFKSKPLN 410

***** ** ******
