# Supplementary material for: Involvement of genes encoding ABI1 protein phosphatases in the response of Brassica napus L. to drought stress
Source: Plant Mol Biol. 2015 Jun 10;88(4-5):445–57. doi: 10.1007/s11103-015-0334-x (PMC4486095; doi:10.1007/s11103-015-0334-x)
Supplement: Supplementary file 7 — Alignments of (A) BnaA01.ABI1.a (B. napus), BraA01.ABI1.a (B. rapa) and BolC01.ABI1.a (B. oleracea) genomic DNA sequences; (B) BnaC07.ABI1.b (B. napus), BraA03.ABI1.b (B. rapa) and BolC07.ABI1.b (B. oleracea) genomic DNA sequences. These sequences were obtained using the same BnaABI1 gene-specific primer pair. Introns are shown in gray. The names of B. napus and B. rapa, and B. napus and B. oleracea, orthologous sequences are marked in red. Asterisks indicate identical residues (DOC 33 kb) [file 11103_2015_334_MOESM7_ESM.doc]

Article title: Involvement of genes encoding ABI1 protein phosphatases in the response of *Brassica napus* L. to drought stress

Journal name: Plant Molecular Biology

Author name: Danuta Babula-Skowrońska, Agnieszka Ludwików, Agata Cieśla, Anna Olejnik, Teresa Cegielska-Taras, Iwona Bartkowiak-Broda, Jan Sadowski

Corresponding authors: Danuta Babula-Skowrońska, Institute of Plant Genetics, Polish Academy of Sciences, Strzeszyńska 34, 60-479 Poznań, Poland; e-mail: dbab@igr.poznan.pl;

Jan Sadowski, Department of Biotechnology, Institute of Molecular Biology and Biotechnology, Faculty of Biology, Adam Mickiewicz University, Umultowska 89, 61-614 Poznań, Poland; e-mail: jsad@amu.edu.pl

A.

*BnaA01.ABI1.a* GTTGGAGAAGAGATCAACGGCTCAGATGAGAGATCGAAGAAGATGATAAGCAGAACAGAG 60

*BraA01.ABI1.a* GCTGGAGAAGAGATCAACGGCTCAGATGAGAGATCGAAGAAGATGATAAGCAGAACAGAG 60

*BolC01.ABI1.a* GCTGGAGAAGAGATCAATGGCTCAGATGAGAGATCGAAGAAGATGATAAGCAGAACAGAG 60

* *************** ******************************************

*BnaA01.ABI1.a* AGCAGGAGTCTGTTCGAGTTCAAGAGTGTGCCTTTGTACGGTGTGACTTCGATCTGTGGG 120

*BraA01.ABI1.a* AGCAGGAGTCTGTTCGAGTTCAAGAGTGTGCCTTTGTACGGTGTGACTTCGATCTGTGGG 120

*BolC01.ABI1.a* AGCAGGAGTCTGTTCGAGTTCAAGAGTGTGCCTTTGTACGGTGTGACTTCGATCTGTGGG 120

************************************************************

*BnaA01.ABI1.a* AGGAGACCGGAGATGGAAGACGCTGTCTCCACGATACCGAGGTTCCTCCAATCTCCGACA 180

*BraA01.ABI1.a* AGGAGACCGGAGATGGAAGACGCTGTCTCCACGATACCGAGGTTCCTCCAATCTCCGACA 180

*BolC01.ABI1.a* AGAAGACCGGAGATGGAAGATGCTGTCTCCACGATACCGAGGTTCCTTCAGTCTCCGACA 180

** ***************** ************************** ** *********

*BnaA01.ABI1.a* AACTCGATGTTAGACGGTCGGTTCAATCCTCAGACAACCGCTCATTTCTTCGGTGTCTAC 240

*BraA01.ABI1.a*  AACTCGATGTTAGACGGTCGGTTCAATCCTCAGACAACCGCTCATTTCTTCGGTGTCTAC 240

*BolC01.ABI1.a* AACTCGATGTTAGACGGTCGGTTCAATCCTCAGACAACCGCTCATTTCTTCGGTGTCTAC 240

************************************************************

*BnaA01.ABI1.a* GATGGTCACGGCGGGTCTCAGGTAATCATCGATGGATCTCCTCTGTTTAATTCAAAGGAT 300

*BraA01.ABI1.a* GATGGTCACGGCGGGTCTCAGGTAAACATCGATGGATCTCCTCTGTTTAATTCAAAGGAT 300

*BolC01.ABI1.a* GACGGTCACGGCGGGTCTCAGGTAAACATCGATGGTTCTCCTCTGTTGATTTC--AGATT 298

** ********************** ********* *********** * *** ** *

*BnaA01.ABI1.a* GGATCTTGATTGGTCTTTGTGT--------------TTGTGTAGGTGGCGAACTATTGTA 346

*BraA01.ABI1.a* GGATCTTGATTGGTCTTTGTGT--------------TTGTGTAGGTGGCGAACTATTGTA 346

*BolC01.ABI1.a* CGATCT-GACAAGTTATTGCGTAAAAAGCTGAGATTTTGTGTAGGTGGCGAACTACTGCA 357

***** ** ** *** ** ******************* ** *

*BnaA01.ABI1.a* GAGAGAGGATGCATTTGGCTTTAGCGGAGGAGATTGCGAAGGAGAAGCCGATGCTCTGCG 406

*BraA01.ABI1.a* GAGAGAGGATGCATTTGGCTTTAGCGGAGGAGATTGCGAAGGAGAAGCCGATGCTCTGCG 406

*BolC01.ABI1.a* GAGAGAGGATGCATTTGGCTTTGGCGGAGGAGATTGCGAAGGAGAAGCCGATGCTCTGCG 417

********************** *************************************

*BnaA01.ABI1.a* ATGGTGACACGTGGCAGGAGAAGTGGAAGAAGGCTTTGTTTAACTCGTTTCTCCGCGTTG 466

*BraA01.ABI1.a* ATGGTGACACGTGGCAGGAGAAGTGGAAGAAGGCTTTGTTTAACTCGTTTCTCCGCGTTG 466

*BolC01.ABI1.a* ATGGTGACACGTGGCAGGAGAAGTGGAAGAAGGCTTTGTTCAATTCGTTCCTTCGCGTTG 477

**************************************** ** ***** ** *******

*BnaA01.ABI1.a*  ACTCGGAGGTGGAGTCGGTCGCGCCGGAGACTGTTGGGTCAACGTCGGTGGTTGCGGTAG 526

*BraA01.ABI1.a*  ACTCGGAGGTGGAGTCGGTCGCGCCGGAGACTGTTGGGTCAACGTCGGTGGTTGCGGTAG 526

*BolC01.ABI1.a* ACTCGGAGGTGGAGTCGGTCGCGCCGGAGACTGTTGGGTCAACGTCGGTGGTTGCCGTTG 537

******************************************************* ** *

*BnaA01.ABI1.a* TTTTCCCGACTCATATCTTTGTAGCTAACTGCGGCGACTCCAGAGCCGTTCTTTGCCGCG 586

*BraA01.ABI1.a* TTTTCCCGACTCATATCTTTGTAGCTAACTGCGGCGACTCCAGAGCCGTTCTTTGCCGCG 586

*BolC01.ABI1.a* TTTTCCCGACTCATATCTTTGTAGCTAACTGCGGTGACTCCAGAGCCGTTCTTTGCCGCG 597

********************************** *************************

*BnaA01.ABI1.a* GCAAAACTGCGCTTCCGTTGTCGACTGACCACAAAGTAAGCTATTTTTCCGACAAAAACA 646

*BraA01.ABI1.a* GCAAAACTGCGCTTCCGTTGTCGACTGACCACAAAGTAAGCTATTTTTCCGACAAAAACA 646

*BolC01.ABI1.a* GCAAAACTGCGCTTCCGTTGTCGACTGACCACAAAGTAATCTATTTT-CCGACAAAACCA 656

*************************************** ******* ********* **

*BnaA01.ABI1.a* GTTTGGATCTCTGTTAAGATTGGTTGTGTCAAAATGTTGAATTTAATTTAATTTCATTTT 706

*BraA01.ABI1.a* GTTTGGATCTCTGTTAAGATTGGTTGTGTCAAAATGTTGAATTTAATTTAATTTCATTTT 706

*BolC01.ABI1.a* GTTTGGATCTTTGGTTAGATTGGTTGTGTCAAGATCTTGAATTTAA-------------- 702

********** ** * **************** ** **********

*BnaA01.ABI1.a* ATTTTACAAAAAAAAAGATTTTGAATCGTTTTCTGATGTGTGTTTTGTGTAATGTGTATA 766

*BraA01.ABI1.a* ATTTTACAAAAAAAAAGATTTTGAATCGTTTTCTGATGTGTGTTTTGTGTAATGTGTATA 766

*BolC01.ABI1.a* ---------------AGAT-----------TACTGATGTATGTTT-GTGTAATGTGCATA 735

**** * ******* ***** ********** ***

*BnaA01.ABI1.a* GCCGGATAGAGAAGATGAAGCGGCGAGGATTGA 799

*BraA01.ABI1.a* GCCGGATAGAGAAGATGAAGCGGCGAGGATTGA 799

*BolC01.ABI1.a* GCCGGATAGAGAAGATGAAGCGGCGAGGATTGA 768

*********************************

B.

*BnaC07.ABI1.b* GATTCCGACAACGGCGA---GACTTCTTCGTGTTCTGTTTCTGGAGCTCAATCTAGAATA 57

*BolC07.ABI1.b* GATTCCGACAACGGCGA---GACTTCTTCGTGTTCTGTTTCTGGAGCTCAATCTAGAATA 57

*BraA03.ABI1.b* GATTCCGACAACGGCGAAGAGACTTCTTCGTGTTCTGTTTCTGGAGCTCAACCTAGGATA 60

***************** ******************************* **** ***

*BnaC07.ABI1.b*  GTTTCGGCATCATCATCATCAT---------CCGGAGAAGGGATCAACGGCTCGGACGAG 108

*BolC07.ABI1.b*  GTTTCGGCATCATCATCATCAT---------CCGGAGAAGGGATCAACGGCTCGGACGAG 90

*BraA03.ABI1.b* GTTTCGTCATCGTCGTCGTCAGACAAAGTCGCCGGAGAAGGGATCAACGGCTCGGACGAG 120

****** **** ** ** ** *****************************

*BnaC07.ABI1.b* AGATCGACGGTTCAGAGCGAGAAGAAGATGATAAGCAGAACGGAGAGCAGGAGCCTGTTC 168

*BolC07.ABI1.b* AGATCGACGGTTCAGAGCGAGAAGAAGATGATAAGCAGAACGGAGAGCAGGAGCCTGTTC 150

*BraA03.ABI1.b* AGATCGACGGTTCAGAGCGAGAAGAAGATGATCAGCAGGACGGAGAGCAGGAGCCTGTTC 180

******************************** ***** *********************

*BnaC07.ABI1.b* GAGTTCAAGAGTGTGCCTTTGTACGGTTTTACTTCGATCTGTGGGAGAAGACCGGAGATG 228

*BolC07.ABI1.b* GAGTTCAAGAGTGTGCCTTTGTACGGTTTTACTTCGATCTGTGGGAGAAGACCGGAGATG 210

*BraA03.ABI1.b* GAGTTCAAGAGCGTGCCTTTGTACGGTTTTACTTCGATCTGTGGGAGAAGACCGGAGATG 240

*********** ************************************************

*BnaC07.ABI1.b* GAAGATGCTGTCTCCGCGATACCTAGGTTCCTTCAATCTCCGACCAATTCGCTGGTGGAT 288

*BolC07.ABI1.b* GAAGATGCTGTCTCCGCGATACCTAGGTTCCTTCAATCTCCGACCAATTCGCTGGTGGAT 270

*BraA03.ABI1.b* GAAGATGCTGTCTCCGCGATACCTAGGTTCCTTCAATCTCCGACCAATTCGCTGGTGGAT 300

************************************************************

*BnaC07.ABI1.b* GGTCGGTTCAATCCTCAGTCAACCGCTCATTTCTTCGGTGTCTACGACGGACACGGCGGA 348

*BolC07.ABI1.b* GGTCGGTTCAATCCTCAGTCAACCGCTCATTTCTTCGGTGTCTACGACGGACACGGCGGT 330

*BraA03.ABI1.b* GGTCGGTTCAATCCTCAGTCAACCGCTCATTTCTTCGGTGTCTACGACGGCCACGGCGGT 360

************************************************** ********

*BnaC07.ABI1.b* TCTCAGGTAAAAAAAA--AAGTCAACGGTCTCCCGAGTATATTCCGACCAGATCCGATAA 406

*BolC07.ABI1.b* TCTCAGGTAAAAAAAA--AAGTCAACGGTCCCCCGAGTAAATGCCGACCAGATCCGATAA 390

*BraA03.ABI1.b* TCTCAGGTTAAAAAAAGAAAGTCAACGGTCTCCTAAGTAGATTCCGACCAGATCCGATAA 418

******** ******* ************ ** **** ** *****************

*BnaC07.ABI1.b* TTTAGTATAAAA-GTCAACCGCTTTG------TGTTGTGTTGTGTTGTGTAGGTTGCGGA 459

*BolC07.ABI1.b* TTTAGTATAAAA-GTCAACCGCTTTG------TGTTGTGTTGTGTTGTGTAGGTTGCGGA 438

*BraA03.ABI1.b* TTTAGTAGAAAAAGTCAACCACTTTGGGGTTGTGTTGTGTTGT-----GTAGGTAGCGGA 478

******* **** ******* ***** *********** ****** *****

*BnaC07.ABI1.b* GTATTGTAGAGAGAGGATGCATCTGGCTTTAGCTGAGGAGATGGCCAGGGAGAATCCGAC 519

*BolC07.ABI1.b*  CTATTGTAGAGAGAGGATGCATCTGGCTTTAGCTGAGGAGATGGCCAGGGAGAATCCGAC 498

*BraA03.ABI1.b* CTATTGTAGAGAGAGGATGCATCTGGCTTTAGCTGAGGAGATAGCCAGGGAGAATCCGAT 538

***************************************** ****************

*BnaC07.ABI1.b* GCT 522

*BolC07.ABI1.b* GCT 501

*BraA03.ABI1.b* GCT 541

***
