# Supplementary material for: Involvement of genes encoding ABI1 protein phosphatases in the response of Brassica napus L. to drought stress
Source: Plant Mol Biol. 2015 Jun 10;88(4-5):445–57. doi: 10.1007/s11103-015-0334-x (PMC4486095; doi:10.1007/s11103-015-0334-x)
Supplement: Supplementary file 9 — Sequence alignment of BnaA01.ABI1.a and BnaC07.ABI1.b fragment genomic DNA including exon I and intron I. Introns are shown in gray. Asterisks indicate identical residues. Translation initiation codons (ATG) are shown (DOC 24 kb) [file 11103_2015_334_MOESM9_ESM.doc]

Article title: Involvement of genes encoding ABI1 protein phosphatases in the response of *Brassica napus* L. to drought stress

Journal name: Plant Molecular Biology

Author name: Danuta Babula-Skowrońska, Agnieszka Ludwików, Agata Cieśla, Anna Olejnik, Teresa Cegielska-Taras, Iwona Bartkowiak-Broda, Jan Sadowski

Corresponding authors: Danuta Babula-Skowrońska, Institute of Plant Genetics, Polish Academy of Sciences, Strzeszyńska 34, 60-479 Poznań, Poland; e-mail: dbab@igr.poznan.pl;

Jan Sadowski, Department of Biotechnology, Institute of Molecular Biology and Biotechnology, Faculty of Biology, Adam Mickiewicz University, Umultowska 89, 61-614 Poznań, Poland; e-mail: jsad@amu.edu.pl

*BnaA01.ABI1.a* ATGGAGGAAGTATCACCAGCCGTTGCTATGCCTTTCATGCCGTTCCCTGAA---CAGCAG 57

*BnaC07.ABI1.b* ATGGAGGAAGCATCTCCGGCGGTTGCTATGCCTTTCATGCCATTCCCCGAGACCCCCCAG 60

********** ** ** * ********************* ***** ** * ***

*BnaA01.ABI1.a* ATGGAGTTAGCAGGGATCATGTTGGGTAAAGGCTACTGCAACGGTCAATACTCGTCTCAG 117

*BnaC07.ABI1.b* ATGGAGCTCGCAGGGATCATGCTGGGTAAAGGCTACTGCAACGGCCAATACTCAGCTCAA 120

** *** * ************ ********************** ******** ****

*BnaA01.ABI1.a* GATTCGGAGAACGGC---------------------TCGTGCTCTGTTTCTG------G 149

*BnaC07.ABI1.b* GATTCCGACAACAACGGCGAGACTT---------CTTCGTGTTCTGTTTCTGGAGCTCA 170

***** ** ** * * ***** **********

*BnaA01.ABI1.a* GTCTAGGAAAGTTTTGACCTCGCGGATCAACTCACCTAACTTGAACATGAAGAAGGAACC 209

*BnaC07.ABI1.b* ATCTAGAATAGTTTCGGC---------------------------------------ATC 191

***** * ***** * * * *

BnaA01.ABI1.a ATCATCATCATCATCATCATCATCATCATCGTCTTCGTCGTCGTCGGAGATAGTTGT--- 266

*BnaC07.ABI1.b* A------------------------------------TCATCATCAT---------C--- 203

* ** ** **

*BnaA01.ABI1.a* TGGAGAAGAGATCAACGGCTCAGATGAGAGATCGA---------------AGAAGATGAT 311

*BnaC07.ABI1.b* CGGAGAAGGGATCAACGGCTCGGACGAGAGATCGACGGTTCAGAGCGAGAAGAAGATGAT 263

******* ************ ** ********** **********

*BnaA01.ABI1.a* AAGCAGAACAGAGAGCAGGAGTCTGTTCGAGTTCAAGAGTGTGCCTTTGTACGGTGTGAC 371

*BnaC07.ABI1.b* AAGCAGAACGGAGAGCAGGAGCCTGTTCGAGTTCAAGAGTGTGCCTTTGTACGGTTTTAC 323

******************************************************* * **

*BnaA01.ABI1.a* TTCGATCTGTGGGAGGAGACCGGAGATGGAAGACGCTGTCTCCACGATACCGAGGTTCCT 431

*BnaC07.ABI1.b* TTCGATCTGTGGGAGAAGACCGGAGATGGAAGATGCTGTCTCCGCGATACCTAGGTTCCT 383

********************************* ********* ******* ********

*BnaA01.ABI1.a* CCAATCTCCGACAAACTCGATGTTAGACGGTCGGTTCAATCCTCAGACAACCGCTCATTT 491

*BnaC07.ABI1.b* TCAATCTCCGACCAATTCGCTGGTGGATGGTCGGTTCAATCCTCAGTCAACCGCTCATTT 443

*********** ** *** ** * ** ****************** *************

*BnaA01.ABI1.a* CTTCGGTGTCTACGATGGTCACGGCGGGTCTCAGGTAATCATCGA--TGGA---TCTCC 545

*BnaC07.ABI1.b* CTTCGGTGTCTACGACGGACACGGCGGATCTCAGGTAAAAAAAAAAGTCAACGGTCTCC 502

*************** ** ******** ********** * * * * *****

*BnaA01.ABI1.a* TCTGTTTAATTCAA---------AGGATGGATCTTGATTGGTCT------TTGTGT-- 586

*BnaC07.ABI1.b* CGAGTATATTCCGACCAGATCCGATAATTTAGTATAA-AAGTCAACCGCTTTGTGTTG 559

** ** * * * * ** * * * ** ******

*BnaA01.ABI1.a* -------TTGTGTAG 594

*BnaC07.ABI1.b* TGTTGTGTTGTGTAG 574

********
