# Supplementary material for: Involvement of genes encoding ABI1 protein phosphatases in the response of Brassica napus L. to drought stress
Source: Plant Mol Biol. 2015 Jun 10;88(4-5):445–57. doi: 10.1007/s11103-015-0334-x (PMC4486095; doi:10.1007/s11103-015-0334-x)
Supplement: Supplementary file 11 — Alignment of BnaA01.ABI1.a and BnaC07.ABI1.b promoter regions. The six CNS (Conserved Non-Coding Sequence) regions were identified using VISualization Tool for Alignments (Vista) tools mVista and rVista (http://rvista.dcode.org/) with sequence similarity at the level of >70% over a 90 bp region; they are highlighted in red. The putative regulatory elements identified by public database searches are boxed in green and named above the core sequence. Gaps for optimal alignment are indicated by dashes. Asterisks beneath sequences indicate identical nucleotide residues (DOC 48 kb) [file 11103_2015_334_MOESM11_ESM.doc]

Article title: Involvement of genes encoding ABI1 protein phosphatases in the response of *Brassica napus* L. to drought stress

Journal name: Plant Molecular Biology

Author name: Danuta Babula-Skowrońska, Agnieszka Ludwików, Agata Cieśla, Anna Olejnik, Teresa Cegielska-Taras, Iwona Bartkowiak-Broda, Jan Sadowski

Corresponding authors: Danuta Babula-Skowrońska, Institute of Plant Genetics, Polish Academy of Sciences, Strzeszyńska 34, 60-479 Poznań, Poland; e-mail: dbab@igr.poznan.pl;

Jan Sadowski, Department of Biotechnology, Institute of Molecular Biology and Biotechnology, Faculty of Biology, Adam Mickiewicz University, Umultowska 89, 61-614 Poznań, Poland; e-mail: jsad@amu.edu.pl

BnaA01.ABI1.a ACTTCGTCCCTTAATCGCGTTGCCCTCAAGGAAAGCTAAAAGATTAACATAATAAATAAG 60

BnaC07.ABI1.b ------------------------------------------------------------

MYB

**TAACCA**

BnaA01.ABI1.a CAAATATTAACCAAAATATCCACTATCCATTTTCTTGGCCATCTCCTCATTTTTTTGTTT 120

BnaC07.ABI1.b ------------------------------------------------------------

MYC

**ACAT**

BnaA01.ABI1.a CATTTAAGCATATGTTGATTTCCAAATATTTTATCTACTTTTTATTTTATTTACCATAAA 180

BnaC07.ABI1.b ------------------------------------------------------------

BnaA01.ABI1.a TAGTTGATGATTGGTTCATTGAATCTGAGGTGTCTTGGGTATTTTGTCTGTTGCCCATCA 240

BnaC07.ABI1.b ------------------------------------------------------------

BnaA01.ABI1.a TCCAGAACCAACTTTCACATTTCCATTTACAGATAACATTTCGTTTACACATTATCCAAA 300

BnaC07.ABI1.b ------------------------------------------------ACATTGTCCAAA 12

***** ******

DPBF NAC

**ACACAAG TACG**

BnaA01.ABI1.a G--AATACGACACAAGTGGCATAAAACATACGGAACTTGGTAAAA-TA-GTTTTATA--- 353

BnaC07.ABI1.b AGAAATACAGCACAAGTGGCACAAAACATATGGAACTTGATAAAAATATGTTGTATACTA 72

***** *********** ******** ******** ***** ** *** ****

BnaA01.ABI1.a GTAACGTTCTGTTATGCACAAAAAAAAGTAACGTCAACCAATATAATGTTATTTATTTGA 413

BnaC07.ABI1.b GTAACGTTCTGTT-------------------GACAACCAATAATAT-TTATTTATTTGA 112

************* * ********* ** ************

**AACGTT**

ACGTTBOX

BnaA01.ABI1.a GATATTAAGAACCAAAAGAATAGGAAAATAAATAAAACA-AGAG---AAAGACAAGATAT 469

BnaC07.ABI1.b GATATTAAGATTC-------TAGAAAAAAAAATAAGAGAGAGAGTTTAAAGACAAGATAT 165

********** * *** **** ****** * * **** *************

NAC

**TACG**

BnaA01.ABI1.a TTACGGAATTAAAAGGTTTGTCTTTGTCAGTGGTGTAATGAACCTTTCTTTCTCTCCTCT 529

BnaC07.ABI1.b TTACGGAATTTAATGGGTTGTCTTTGTCAGTG---CAATGAATCTTT-TTTCTCTCGTCT 221

********** ** ** *************** ****** **** ******** ***

**TACG**

NAC

ABRE

**TACGGTC** SALT

SALT LTRE ABRE **GGTG** DRE

**GTGG TCCGACC ACGTG ACCGAGA**

BnaA01.ABI1.a CTCCCGCCACCCAACTAACACATCTCCGACCGTACACGTCACCCCCTCTCGGTCACCAAA 589

BnaC07.ABI1.b ----------------AACACATCTCGAACCGTACACGTCACACTCTCTCGTTCACCAAA 265

********** ************** * ****** ********

**GACGTGTA**

ABRE

ABRE

**CTATACGTGTCCC**

BnaA01.ABI1.a ACTGGACTTTTCTTCCTATACGTGTCCCTCATC-GCCGGTTT-CTGTTAAACATGGGGCC 647

BnaC07.ABI1.b ATAGACTTTTTCTTCCTCTACCTGTCGATCATCCACCGGTTTACCGCTGAACGTGGGACC 325

* * ********** *** **** ***** ******* * * * *** **** **

BnaA01.ABI1.a CAATGTTATTCATATT-TTTTTTTCTTAAATTTATAGATTTTAAAATATTGACGGATTCT 706

BnaC07.ABI1.b -ATTATTATTCACGTTATTTTTTTTTTTGA---ACAACATTCACGTTATT------TTCT 375

* * ******* ** ******* ** * * * ** * **** ****

BnaA01.ABI1.a CTC-ATGTTCTTGGTTCATTGCT------CAATTAT-TTCCATGATGAGTCCTATCATAA 758

BnaC07.ABI1.b TTCTATGATTTTTGTTCTTTATTGTGATACAAATATGTTCCAT-ACAAATAGTTTTGT-- 432

** *** * ** **** ** * *** *** ****** * * * * * *

LTRE

**TCCGACT**

BnaA01.ABI1.a TGTTCAGTCGGAATCATGTTTTTATTTTCTGTA-TTGCGACAACAACAACAACATGTTGT 817

BnaC07.ABI1.b TTTCTGGTCA-AAT--GGTCAATACAAAGAGTAATTAGGGTCGAATTAATGGATTTCTAT 489

* * *** *** ** ** *** ** * * ** * * *

NAC

**CACG**

ABRE

**ATCTACGTGCATT**

BnaA01.ABI1.a GGAAAAGAAATGAAAAGAAAATGAATGCACGTAGATTTTAACGTATTTCGTGTAAATTTA 877

BnaC07.ABI1.b C-AAAACATCTCGAAATAAAATGATTGCAAGTAGGTTTTAACCCATTT----------TA 538

**** * * *** ******* **** **** ******* **** **

BnaA01.ABI1.a TAATTGCAAGACCGATTCT--TCACTTCATATTCTCAGGAAAATAC------CAATTTCT 929

BnaC07.ABI1.b TAATTGCGAGACCGGTTATATTTTCGTGATGTTTACAGAAAAATACAAAGACCAAATTCT 598

******* ****** ** * * * * ** ** *** ******* *** ****

MYB

**TAACCA**

BnaA01.ABI1.a TTCAGCTCTGCATAACCATT-----------------TCA-----TGGC----------- 956

BnaC07.ABI1.b TTCTGTTGTGCAGAACCATTCTTTTTTTTTTTTTTGTTCACCATTTTGCAGAACCATTCT 658

*** * * **** ******* *** * **

BnaA01.ABI1.a ---TACATAAATTTTAAAAATAATTAAGATTGAATAATCTAGGTTATGGTGGTTGTATGT 1013

BnaC07.ABI1.b ATATATATGGATTTTTAGTGGATTAAAGATTCAATTATATAGGTTATGAATATAGTTTGT 718

** ** ***** * * * ****** *** ** ********* * ** ***

BnaA01.ABI1.a TCATATGCTAACATATACATACAAACAATGCTTTTAGGTTTTAAGTTTGACCAAAAATAG 1073

BnaC07.ABI1.b TCCTACGCTAACAGATATATACAAA-AATA-------GTTTGAAGTTTGACCAAAAAAGG 770

** ** ******* *** ******* *** **** *************** *

BnaA01.ABI1.a TTTCTAAGATATATAGTTTTAGATTATTTGACTAATGATTCCCATTAAATTATAATTATT 1133

BnaC07.ABI1.b TTTACAAATTATAAAAAT---GATAATGT----AATGATTCCCCTTAAATTATAATTATT 823

*** ** **** * * *** ** * ********** ****************

BnaA01.ABI1.a CAGATATCATACAGGCTGTCTTCCATTTTTACAGAATATAAGAACATCCAATTCT----- 1188

BnaC07.ABI1.b AAGATATTTTACCGGTGGTCTTCTATTTTTCCAGAAAATAAGAACATCCAATTCTTTTCT 883

****** *** ** ****** ****** ***** ******************

BnaA01.ABI1.a -----------------------ATTATTCTTATACTGAAAATGACAGAAGTTT-AATTG 1224

BnaC07.ABI1.b TATCTTAATTATACGGATAAAATATTATTCTTATACTGAAAATGACAGAAGTTTTGATTA 943

******************************* ***

BnaA01.ABI1.a TTTTTTAATATA---TCTA-----TATATGAGTATGTT----------TTTAGTCCAATG 1266

BnaC07.ABI1.b TTATTTTGTTTAATTTTTAAATGGTATACGAATATGATGTTTGGTTTATTTAGTCCAAAG 1003

** *** * ** * ** **** ** **** * ********** *

**AAACCA**

MYB

BnaA01.ABI1.a TTTAGAAGTTTCTAATAAACTTT------------GTTAAAT----------GACAAATA 1304

BnaC07.ABI1.b GTTAGAAGTTACGAATAAACTTTCGTGTATTTCGGATTAAATCTCTATATGTGACAAATA 1063

********* * ********** ****** ********

**CACG**

NAC (ANAC019, ANAC06)

motif A of ABRE

**TACGTGTC**

BnaA01.ABI1.a AATATTTTGTAAACTAC--GACTTAAATCCGAATGTGACACGTATGTCTATTAGTGAAAG 1362

BnaC07.ABI1.b AATATATTGTAAATTACAAGACTTAAATCGGAATGTGACACGTACGTCTATTAGTGAAAG 1123

***** ******* *** ********** ************** ***************

**TACGTGTC**

motif A of ABRE

MYB

**AGTT**

BnaA01.ABI1.a ACATCTAATAAAAC-------TACATTTAATTCATTAAACCGATGACCTAACTAAAGTTT 1415

BnaC07.ABI1.b TCATCTACTAAAATATACTAGTACAATAATTTCATTAAACCGAATGCTTAACTAAAGTT- 1182

****** ***** **** * * ************* * ***********

**AGTT**

MYB

BnaA01.ABI1.a AGAAGCTAAACGGAGTATTTCT----------TCTAACAAG---------AAAA------ 1450

BnaC07.ABI1.b AATGATTAAACAAAATTTTTTTGGTGAAATGATTAAACAAATTTCGTTGTGAAATTCTCT 1242

* ***** * * *** * * ***** ***

BnaA01.ABI1.a ------AAGAATTATT---TGGTGTA----------------------ATAATATTTG-- 1477

BnaC07.ABI1.b TAATTCAATGATTACTAAATACTGTATTAAATATTGAAGATAACGAGAATAAAATTTGTT 1302

** **** * * **** **** *****

BnaA01.ABI1.a ----------ATGATT-----------------------CAA-------CATTTATT--- 1494

BnaC07.ABI1.b TTATAAATTAATGGTTTATAAAGAAGTTTTAACGTAATGCAAATAAAACCATTCATTAAA 1362

*** ** *** **** ***

**AAACCA AAACCA**

MYB MYB

BnaA01.ABI1.a -TGTACAA----AGTTA----------TTA-----AAAGTGAAA---------------- 1518

BnaC07.ABI1.b TTGTAAAATTCTGGTTAGATAGATAGTTTATCTGTGAAGTTAAATTTTTTGTTTTCATAG 1422

**** ** **** *** **** ***

**CTAACCA**

MYB

BnaA01.ABI1.a ----------AAAAAGCCG-AGCAATGAATCAAAA---------TAAAATA--------- 1549

BnaC07.ABI1.b TACTTACTCTAAAAATTTGTAATAGAGAATCATAAATTGTTTGATAAAATAACATAGGGT 1482

***** * * * ****** ** *******

BnaA01.ABI1.a ------------------------CAAAAAAAATTATTGAATGCAGAAA-----AG---T 1577

BnaC07.ABI1.b TTTGGTTTTAGAATATTAATCCGGCCGAAAAAACTACC-AATGAAAAAACCTACAGATTT 1541

* ****** ** **** * *** ** *

**AAACCA**

MYB

MYC

**ACAC**

BnaA01.ABI1.a TGGAAAGC---AGGTGTCTTTTTC-TGATTTTAAAATCTCAAGAACGAAACATCTCCCCT 1633

BnaC07.ABI1.b TTGAACACTGAAAACGTATCTTTTATGATTTTAAAATTCCAAGAACGAAACATCTCCCCT 1601

* *** * * ** * *** ************ *********************

BnaA01.ABI1.a TATTACAAAAACAACAAAATAATTAAAA---ACAGA-----------AGTTTTATAATGT 1679

BnaC07.ABI1.b TATTACAAAAAAAAAAAAAAAACTAAAAGGAACAGATAATAGAACATGACTTTATAATAA 1661

*********** ** **** ** ***** ***** ********

BnaA01.ABI1.a AATAAAATAAAGATAAATAATATCTATTCAAGTAATAACATAAATAGCAGATTTGAAGGG 1739

BnaC07.ABI1.b AATAAAATATAGATCAATA-CCTATATTTAAGT--TAACATAAATAGCAGATT------- 1711

********* **** **** * **** **** ******************

BnaA01.ABI1.a ATTATTGCCTCTGCGTTACCTATCTCTCCACTTCCCTTCCCTTCTTTCTTTCTCCTTTTA 1799

BnaC07.ABI1.b ---GTTGCCTTTGCGT-ACCTATATCCTC-----------CTCCTTTTTTTCTCTTTATA 1756

****** ***** ****** ** * ** **** ****** ** **

**TACG**

NAC

BnaA01.ABI1.a TCTGTGAACAAGAAGAA-------------------------AAAAGAGAGCCTTCTTTC 1834

BnaC07.ABI1.b TCTGTGAAGAAGAAGAAGGCGCGTGAAGTATTAGAAGAAGAAAAAAAACAGTCTTCTTTC 1816

******** ******** **** * ** ********

BnaA01.ABI1.a AACTTCTAGGTAGTTGAATCTACAAACAGCATTAGCTGGAAAAATTAATTGAAGAAAGAG 1894

BnaC07.ABI1.b AACTTTTAGGTAGTAGGA------------ATTAATTAAAGAGA-------GAGAGAGAG 1857

***** ******** * * **** * * * * *** ****

BnaA01.ABI1.a AGAGAT------ACAATCTTTAAGAGGCTGTAACGAATTACCCACAACCCAGGAAACCCT 1948

BnaC07.ABI1.b AAATATTTGCACAGAGTCATTAAGAGGTTCTAACGAATTACCCACAATCTAGGAAACCCT 1917

* * ** * * ** ******** * ***************** * **********

BnaA01.ABI1.a -GATAAATATTTGATCT--TTC---CCGGGAAAGTTTTGGTGTATCATGAGAATCTTGTC 2002

BnaC07.ABI1.b CGTTTAAAATTTAATTCAATTCAATCCATTCATCTTTTTTTTTCTTTTTGCTGTGTGGTT 1977

* * ** **** ** *** ** * **** * * * * * * **

BnaA01.ABI1.a TCCATACCTTCTAATAAAGTTGATCTCTTAAATTTTTTCTTGTAGAAATCTGTCGAATTT 2062

BnaC07.ABI1.b --GAT-TTTCCCGGGAAAATTGGTGTATCAT-CTGTTTCCTGCGGTAGC---TAAAAGTT 2030

** * * *** *** * * * * * **** ** * * * ** **

MYB

**AAACCA**

BnaA01.ABI1.a TCGGCTTTTCTTCC-ATTAATAGATCA-AACCATCTT---TACACAACCAAACAACTAAA 2117

BnaC07.ABI1.b CCTTCTTTTTTTCTTGGTGAGGAGTCATGGGCTTCTTGTCTACATAGCT--TCTTCTAAG 2088

* ***** *** * * *** * **** **** * * * ****

BnaA01.ABI1.a AAAAA--------CTCAAACCTTT--ACAAAA---------------CATTTCTG----A 2148

BnaC07.ABI1.b AAAGTTGTTTCTCCTCAAATCTTTTTGTAGAAGATCTGTCGCCTTTTCACCTTTGTTCCA 2148

*** ****** **** * ** ** * ** *

BnaA01.ABI1.a GAAAAAATCAAAAAAAAAACAGAGCATCAATG 2180

BnaC07.ABI1.b TTAAAGCTCAAAGCTTTACGATTCCAACAATG 2180

*** ***** * * ** *****
